# Transient infection of the zebrafish notochord with *E. coli* induces chronic inflammation

**DOI:** 10.1242/dmm.014498

**Published:** 2014-07

**Authors:** Mai Nguyen-Chi, Quang Tien Phan, Catherine Gonzalez, Jean-François Dubremetz, Jean-Pierre Levraud, Georges Lutfalla

**Affiliations:** 1 Dynamique des Interactions Membranaires Normales et Pathologiques, Université Montpellier 2, UMR 5235, case 107, Place Eugène Bataillon, 34095 Montpellier Cedex 05, France.; 2 CNRS; UMR 5235, Place Eugène Bataillon, 34095 Montpellier Cedex 05, France.; 3 Macrophages et Développement de l’Immunité, Institut Pasteur, Paris F-75015, France.; 4 CNRS URA2578, Paris F-75015, France.

**Keywords:** Zebrafish, Neutrophils, Inflammation, Interleukin-1β

## Abstract

Zebrafish embryos and larvae are now well-established models in which to study infectious diseases. Infections with non-pathogenic Gram-negative *Escherichia coli* induce a strong and reproducible inflammatory response. Here, we study the cellular response of zebrafish larvae when *E. coli* bacteria are injected into the notochord and describe the effects. First, we provide direct evidence that the notochord is a unique organ that is inaccessible to leukocytes (macrophages and neutrophils) during the early stages of inflammation. Second, we show that notochord infection induces a host response that is characterised by rapid clearance of the bacteria, strong leukocyte recruitment around the notochord and prolonged inflammation that lasts several days after bacteria clearance. During this inflammatory response, *il1b* is first expressed in macrophages and subsequently at high levels in neutrophils. Moreover, knock down of *il1b* alters the recruitment of neutrophils to the notochord, demonstrating the important role of this cytokine in the maintenance of inflammation in the notochord. Eventually, infection of the notochord induces severe defects of the notochord that correlate with neutrophil degranulation occurring around this tissue. This is the first *in vivo* evidence that neutrophils can degranulate in the absence of a direct encounter with a pathogen. Persistent inflammation, neutrophil infiltration and restructuring of the extracellular matrix are defects that resemble those seen in bone infection and in some chondropathies. As the notochord is a transient embryonic structure that is closely related to cartilage and bone and that contributes to vertebral column formation, we propose infection of the notochord in zebrafish larvae as a new model to study the cellular and molecular mechanisms underlying cartilage and bone inflammation.

## INTRODUCTION

Osteomyelitis, chondritis and septic arthritis are inflammatory diseases of bones, cartilage and joints, respectively, that are often caused by bacterial infections. These diseases are caused by local or systemic immune responses that lead to leukocyte infiltration, damage to extracellular matrix, compression of vasculature and the progressive destruction of bone and joints (Wright and Nair, 2010). The bacteria that are involved are predominantly *Staphylococcus aureus*, *Streptococcus* spp. and members of Gram-negative Enterobacteriaceae, including *Escherichia coli* (Gutierrez, 2005; Lew and Waldvogel, 2004). Although the pathogen is often present within the infected site, it is occasionally undetectable, despite the persistence of immunological symptoms. Substantial evidence supports the importance of innate immunity in the physiopathology of these diseases; however, its exact role is still poorly understood (Lew and Waldvogel, 2004; Wright et al., 2010).

The innate immune response is the first defence of the host in the case of infection. Macrophages and neutrophils are two major effectors of innate immunity against microbes. Neutrophils are the first to be recruited to the infected tissue; they migrate rapidly by responding to gradients of chemoattractant molecules – such as the contents of damaged cells, bacterial products and host chemokines (Deng et al., 2013; Renshaw and Trede, 2012). Macrophages are recruited later in order to aid the removal of dead cells and microbes; they play a role in remodelling the injured tissue and activating adaptive immunity. Neutrophils and macrophages are both professional phagocytes, but the efficiency of phagocytosis depends on the site at which they encounter bacteria (Colucci-Guyon et al., 2011).

Although leukocytes can migrate rapidly and specifically to an infection site, most tissues have specific resident macrophages permanently patrolling the tissue, circulating through tissues to eliminate cellular debris and microorganisms. From this immunological perspective, bone and cartilage are very specific tissues in which, owing to their highly structured and resistant extracellular matrix, no specialised phagocytes patrol. The best-documented example is that of bones. Resident bone macrophages, OsteoMacs, are not present within the bone matrix but, with their stellate morphology, do extensively cover bone surfaces (Pettit et al., 2008). They probably act as guards to prevent microbes from entering or attacking the bone. By contrast, osteoblasts (that make bone) and osteoclasts (monocyte-macrophage lineage cells that resorb bone) are capable of phagocytosis, probably to compensate for the absence of professional phagocytes within the tissue. Some pathogens have even developed specific strategies to colonise these cells (Wright and Nair, 2010). Although documented to a lesser extent, the situation in cartilage is similar, with chondrocytes displaying phagocytic activity and a highly phagocytic cell population that specifically expresses the macrophage marker CD163, present below the superior zone at the periphery of cartilage (**Castillo and Kourí, 2004; Jiao et al., 2013).

To address the complexity of the chronic inflammation of bones and joints, a number of mammalian infection models have been developed (Patel et al., 2009). Most of them rely on the injection of *Staphylococcus aureus*, or other bacteria, directly into the bone in combination with an adjuvant or a trauma. Owing to these models, advances have been made in understanding the disease and in tuning antibiotic therapies; however, to understand the complexity of dynamic interactions during inflammation, non-invasive and tractable models in which pathogens and immune cells can be imaged in the context of three-dimensional tissue architecture in the host are required. Owing to its exceptional transparency, the zebrafish embryo is an exquisite vertebrate model for the study of tissue-specific infections and the inflammatory consequences within a homologous tissue.

**TRANSLATIONAL IMPACT**

**Clinical issue**

Osteomyelitis, chondritis and septic arthritis are inflammatory diseases of bone, cartilage and joints that are often caused by bacterial infections. In these diseases, local or systemic immune responses lead to leukocyte infiltration and the progressive destruction of bone and joints. Special culture cell systems and several mammalian infection models have been developed to understand these diseases and to tune antibiotic therapies. However, the study of the complex dynamic interactions that occur during inflammation requires non-invasive models in which pathogens and immune cells can be imaged in the context of three-dimensional tissue architecture in the host. In the last decade, the zebrafish, owing to the transparency of its embryo and the ease with which it can be genetically manipulated, has emerged as an exquisite system for visualising the immune response to pathogen infections *in vivo* and in real time.

**Results**

Here, the authors develop a zebrafish model to investigate the inflammatory response that is induced by infection of the notochord, a cartilaginous-like embryonic tissue that participates in vertebral column formation and shares many similarities with chondrocytes. Using multidimensional confocal imaging, the authors show that, during the early stages of inflammation, infected zebrafish notochord is inaccessible to leukocytes, even though these professional phagocytes engulf microbes that had been injected into other tissues of the zebrafish embryo. Using *Escherichia coli*, a pathogen that cannot develop within the notochord, the authors show that a transient infection in this tissue triggers a severe and persistent inflammation that is characterised by a massive recruitment of leukocytes, extensive degranulation of neutrophils and leukocyte infiltration into the notochord. Notably, this non-resolved inflammation is accompanied by extracellular matrix remodelling and by the development of notochord lesions and dysmorphic vertebrae development. Finally, the authors report that an Il1b cytokine burst occurs during notochord inflammation that is specifically required for the establishment of the inflammatory episode and of notochord lesions.

**Implications and future directions**

These findings establish this *in vivo* approach as a relevant new vertebrate model of chronic inflammation in matrix-enriched tissues, such as bones and cartilage. Indeed, the symptoms exhibited by the notochord-infected larvae are reminiscent of those seen in bone and cartilage infection in mammals. Furthermore, these findings provide the first *in vivo* evidence that neutrophils can degranulate in the absence of a direct encounter with a pathogen. Thus, transient infection of the zebrafish notochord provides a unique model in which to visualise at high resolution the cellular events underlying pathological inflammation.

At the embryonic and early-larval stages, zebrafish do not possess bone. However, the notochord, a transient embryonic structure that is present in all chordates, in addition to its essential roles in vertebrate patterning during development, serves as an axial skeleton of the embryo until other structures, such as the vertebrae, form (Stemple, 2005). The notochord comprises vacuolated cells that are encased in a sheath of collagen. Osmotic pressure within the vacuoles exerts such a pressure on the thick collagenous sheath that it forms a hydrostatic skeleton in the embryo and young larva. Notochordal cells that express many cartilage-specific genes are most closely related to chondrocytes, but as development proceeds, they become ossified in regions of developing vertebrae and contribute to the formation of the centre of the intervertebral discs in a structure called the nucleus pulposis (Stemple, 2005). The notochord can be studied to dissect the molecular mechanisms that are involved in the immune response to infection in a chrondrocyte-like tissue. Previous results from our laboratory have shown that the injection of an attenuated mutant of *Mycobacterium marinum*, TesA:Tn, into the notochord allows active replication of the bacteria, whereas the infection is easily controlled by professional phagocytes when injected at other locations (Alibaud et al., 2011). The drawback of this infection model is that the active replication of the mycobacteria within the notochord ultimately leads to the death of the host. As neither macrophage nor neutrophil infiltration is observed (by using electron microscopy), we hypothesised that the notochord is an organ that is inaccessible to leukocytes and that this organ could be a good model in which to study the outcome of an infection in a tissue where leukocytes cannot reach the pathogen.

Here, we show that the injection of non-pathogenic Gram-negative bacteria *Escherichia coli* K12 into the notochord of zebrafish embryos induces a strong immune response without compromising the survival of the host. Using a combination of imaging and genetic techniques, we investigate the fate of the bacteria in this organ, analyse the interaction between the bacteria and the leukocytes, describe the inflammation induced by the infection and analyse the consequences of this inflammation.

## RESULTS

### Zebrafish larvae rapidly clear *E. coli* infection in the notochord

**Fig. 1.**
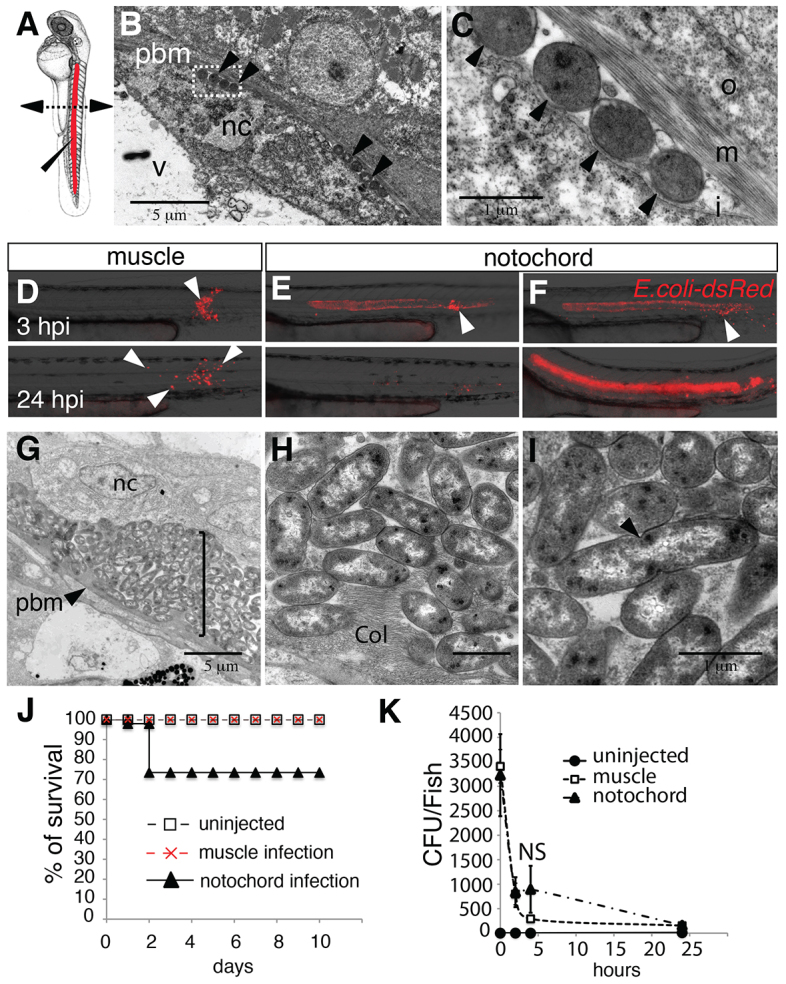
**Injection of *Escherichia coli* into the notochord of zebrafish larvae.** (A) Embryos at 48 hpf. Bacteria were injected (elongated arrowhead) into the notochord (red) dorsal to the urogenital opening. The dashed line with arrowheads shows the region where cross-sections have been performed for electron microscopy. (B,C) Transmission electron microscopy analysis of a larva 15 minutes after injection of bacteria into the notochord; C is a higher magnification of the boxed region in B. Arrowheads indicate bacteria. Nc, notochordal cell; v, vacuole; pbm, peri-notochordal basement membrane that comprises three layers – o, outer; m, medium (collagen); I, inner. (D-F) The fate and distribution of DsRed-expressing *E. coli* (red) after infection of muscle with 3000 CFU (D) or notochord with 2000 CFU (E) and 3000 CFU (F). Each larva was followed and imaged at 3 (top row) and 24 (bottom row) hpi. Arrowheads indicate clusters of bacteria. (G-I) Transmission electron microscopy analysis of the notochord of larvae that had been infected in the notochord (11 hpi); H,I show magnifications of regions of G. (G) Numerous bacteria (bracket) are found between the peri-notochordal basement membrane (pbm, arrowheads) and notochord cells (nc). (H) Collagen (col) disorganisation is observed next to bacteria. (I) Extracellularly replicating bacterium (arrowhead). (J) Survival was scored of uninfected larvae and those that were infected in the muscle or notochord (*n*>24 each). These results are representative of three independent experiments, *P*<0.0005 for notochord injection versus uninjected. (K) Whole embryo bacterial counts of *E. coli* when injected into the muscle or notochord. Results are expressed as the mean number of CFU per larvae±s.e.m. (*n*>5 larvae per timepoint). NS: not significant. Scale bars: 5 μm (B,G); 1 μm (C,H,I).

To characterise the immune response to notochord infection, we first injected 3×10^3^ bacteria of an *Escherichia coli* K12 strain that expressed the fluorescent protein DsRed (*E. coli*-DsRed) into the notochord of zebrafish embryos at 45 hours post-fertilisation (hpf) (Fig. 1A). Injection through the collagen sheath, just posterior to the yolk extension, leads to the anterior sliding of the bacteria around the notochordal cells inside of the collagen sheath. Fifteen minutes after injection, the embryos were fixed, and the localisation of the bacteria was checked by using transmission electron microscopy (TEM). Bacteria were found inside the notochord, mainly at the periphery, pinned against the collagen sheath by the turgescent notochordal cells (Fig. 1B,C). The fate of the bacteria in the notochord was monitored by live-imaging microscopy and compared to the fate of bacteria that were injected inside muscles. When injected into muscles, bacteria were restricted to the injected somite for the first hours, but after 24 hours, their fluorescent remnants were observed within motile cells that migrated away from the site of injection (Fig. 1D). By contrast, in most cases (73%, 57 out of 78), the day following injection into the notochord, fluorescent bacteria were not observed in the notochord, suggesting that bacteria had been removed (Fig. 1E). In the other 27% (21 out of 78) – presumably those embryos that received the higher bacterial load – bacteria proliferated within the notochord (Fig. 1F–I) and led to larval death within 3 days, as illustrated by the reduced survival of the embryos in which bacteria had been injected into the notochord (Fig. 1J). Further experiments were performed using lower bacterial loads (2×10^3^); at this load, all embryos survived and bacterial division was not observed (by using TEM; data not shown). The clearance of bacteria from the notochord of most of the infected fishes was confirmed by counting colony-forming units (CFUs) (Fig. 1K), indicating that bacteria, if not too numerous, are efficiently cleared from the notochord within the first 24 hours of infection.

### Neutrophils and macrophages fail to reach, and engulf, bacteria in the notochord

**Fig. 2.**
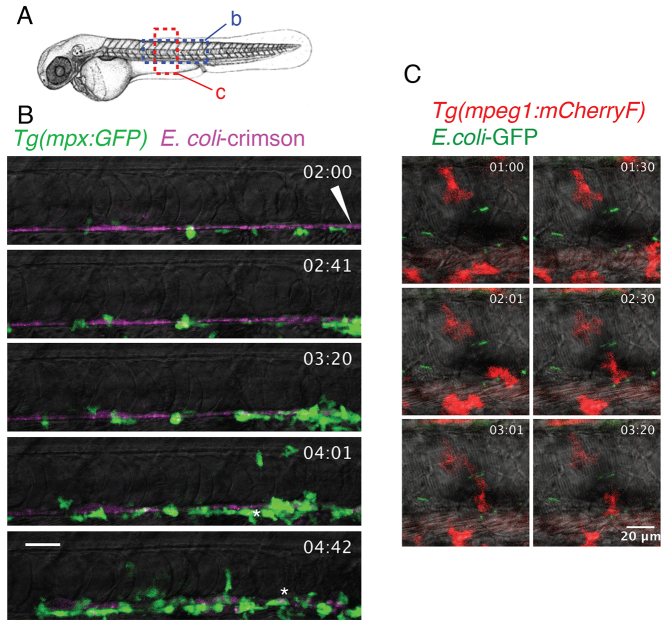
**Behaviour of neutrophils and macrophages following notochord infection.** (A) Diagram showing the regions of the larvae imaged in B and C (dashed boxes). (B) *Tg*(*mpx:GFP*) larvae that had been infected with crimson-expressing *E. coli* (magenta) in the notochord at 48 hpf. The general behaviour of neutrophils (green) was imaged using four-dimensional confocal microscopy, starting at 2 hpi for a duration of 3 hours (the time post-infection is shown in the top right corner). *Neutrophils loaded with bacteria, the arrowhead indicates the injection site. (C) *Tg*(*mpeg1:mCherryF*) larvae that had been infected with GFP-expressing *E. coli* (green) at 48 hpf in the notochord were imaged in the region represented in A to visualise the behaviour of macrophages (red) using four-dimensional confocal microscopy at 1 hpi for a duration of 3 hours and 20 minutes. Scale bars: 20 μm.

During the first weeks of development, the zebrafish larva has no functional adaptive immune system, relying mostly on macrophages and neutrophils, to fight infections (Lieschke et al., 2001; Meijer and Spaink, 2011; Renshaw and Trede, 2012). Therefore, we determined whether these cells were able to reach the bacteria in the notochord in order to phagocytose and/or kill them. To study neutrophil-microbe interactions, we injected fluorescent *E. coli* into *Tg*(*mpx:GFP*) embryos, which expressed green fluorescent protein (GFP) under the control of the neutrophil-specific promoter of *myeloperoxidase* (*mpx*) (Renshaw et al., 2006), and we then imaged neutrophil behaviour by using confocal time-lapse microscopy from 2 hours post-infection (hpi). In contrast to PBS-injected controls (data not shown), neutrophils were primarily attracted to the injection site, then, arriving from the ventral side of the larvae (Fig. 2A,B; supplementary material Movies 1, 2), they crawled along the outside of the infected notochord, spreading towards the anterior region (Fig. 2B; supplementary material Movie 1). Although neutrophils appeared to be attempting to try to phagocytose bacteria (i.e. crawling forward and backward in the vicinity of bacteria, emitting cytoplasmic protrusions), they failed to engulf them (supplementary material Movie 3). Only a few neutrophils contained engulfed bacteria, but these leukocytes were carefully, and retrospectively, traced from the injection site, suggesting that they phagocytosed a few *E.coli* that were deposited at the injury site of the notochord (Fig. 2B; supplementary material Movie 1). This behaviour is in stark contrast with that observed in muscles, where many neutrophils displayed phagosomes that were full of bacteria at 2 hpi and still efficiently engulfed bacteria (data not shown; Colucci-Guyon et al., 2011).

To analyse macrophage-bacteria interactions, we constructed the *Tg*(*mpeg1:mCherryF*) line that expressed red fluorescent macrophages. Transgenic larvae were infected either in the bloodstream or in the notochord, and macrophage behaviour was recorded at 1 hpi. Similar to neutrophils, although less mobile, macrophages were recruited to the infected notochord and extended pseudopodal protrusions in attempts to engulf bacteria (Fig. 2A,C; supplementary material Movies 4, 5). In contrast to the numerous macrophages that were laden with *E. coli* after bloodstream infection (supplementary material Movie 6), macrophages were inefficient at engulfing the bacteria present in the notochord. It is noteworthy that no significant difference in the time to the first recruitment was detected between neutrophils and macrophages (data not shown).

**Fig. 3.**
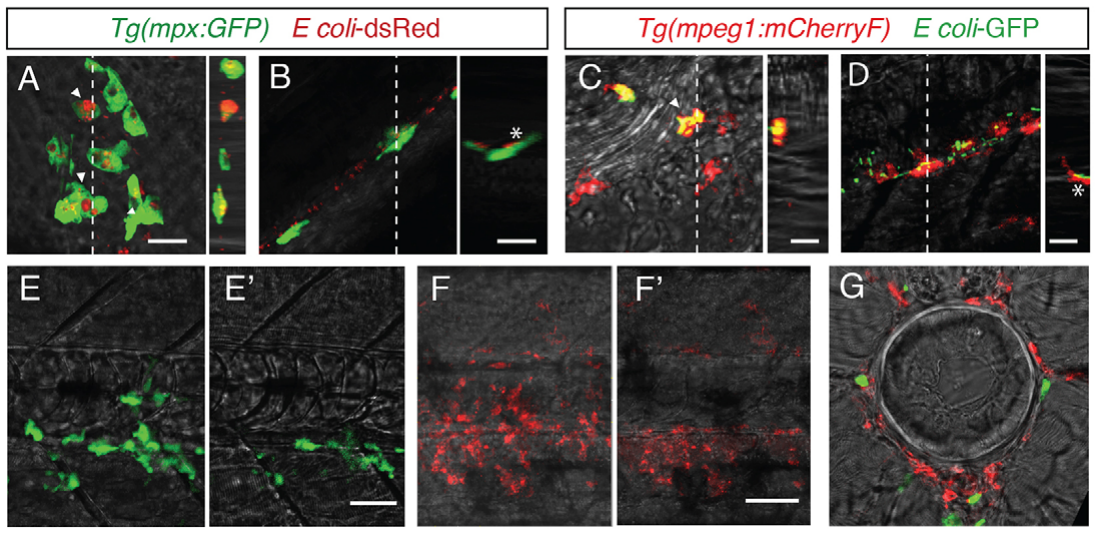
**Leukocytes do not enter the notochord during the early phase of infection.** (A,B) Multiscan confocal analysis at 2 hpi of neutrophils (green) in *Tg*(*mpx:GFP*) larvae after muscle (A) or notochord infection (B) with DsRed-expressing *E. coli*. (C,D) Multiscan confocal analysis of macrophages (red) in *Tg*(*mpeg1:mCherryF*) larvae after intravenous (C) or notochord (D) infection with *E. coli-GFP* (green) at 2 hpi. In A–D, the left panels are *xy* maximum projections and the right panels are *yz* cross-section views at the position of the stippled line. Arrowheads show bacteria-containing leukocytes, *Leukocytes that were located next to bacteria but did not contain any. (E–G) Confocal analysis at 24 hpi of the notochord with fluorescent *E. coli*. Bacteria were no longer visible at 24 hpi. (E,E′) *Tg*(*mpx:GFP*) larva showing neutrophils (green), (F,F′) *Tg*(*mpeg1:mCherryF*) showing macrophages, (E,F) maximum projection, (E′,F′) single confocal scan. (G) Confocal analysis of a 60-μm cross-section from a double-transgenic larva – neutrophils (green) and macrophages (red) accumulated around the notochord but did not enter this structure, as shown. Scale bars: 10 μm (A–D); 20 μm (E–G).

The fact that leukocytes easily engulfed bacteria at the injection site, where the notochordal collagen sheath had been damaged by the injection, but not in the undamaged upstream region suggests that bacteria might not be accessible to leukocytes because of the physical properties of the notochord (data not shown). We tested this hypothesis by performing three-dimensional reconstruction of confocal acquisitions. From 2 hpi onwards, bacteria were found in the phagosomes of neutrophils and macrophages after injection into muscle (Fig. 3A) and the bloodstream, respectively (Fig. 3C); however, they remained next to, but outside of, immune cells after notochord infection (Fig. 3B,D). Indeed, many leukocytes accumulated around the notochord but could not infiltrate it, even at 24 hpi (Fig. 3E–G).

To confirm further that leukocytes could not reach the bacteria, and engulf them, in the notochord, we analysed the ultrastructure of the notochord of infected larvae by using TEM at different timepoints following infection. At 15 minutes post-infection, bacteria were found in the periphery of the notochord, between notochordal cells and the collagen sheath as mentioned above (supplementary material Fig. S1A–C). At 2 hpi and 4 hpi, bacteria remained in the same location – in the extracellular space – although they were less numerous at 4 hpi (supplementary material Fig. S1D–F; data not shown for the 4 hpi timepoint). Replicating bacteria were never observed under these conditions. This correlated with the observation that DsRed fluorescence in *E. coli* remained constant in the notochord for a few hours following injection before diminishing completely. Although a few leukocytes were detected outside of the notochord (data not shown), none were found inside, confirming that leukocytes cannot phagocytose bacteria in this structure (supplementary material Fig. S1). Strikingly, numerous *E. coli* displayed an altered morphology (supplementary material Fig. S1B,C,F), suggesting they are eliminated by a humoral lysis mechanism. No altered morphologies were observed when higher doses of bacteria were used, suggesting that the humoral lysis mechanism is overpowered when the notochord is injected with higher doses of *E. coli*.

### Notochord infection induces a strong and persistent inflammatory reaction

**Fig. 4.**
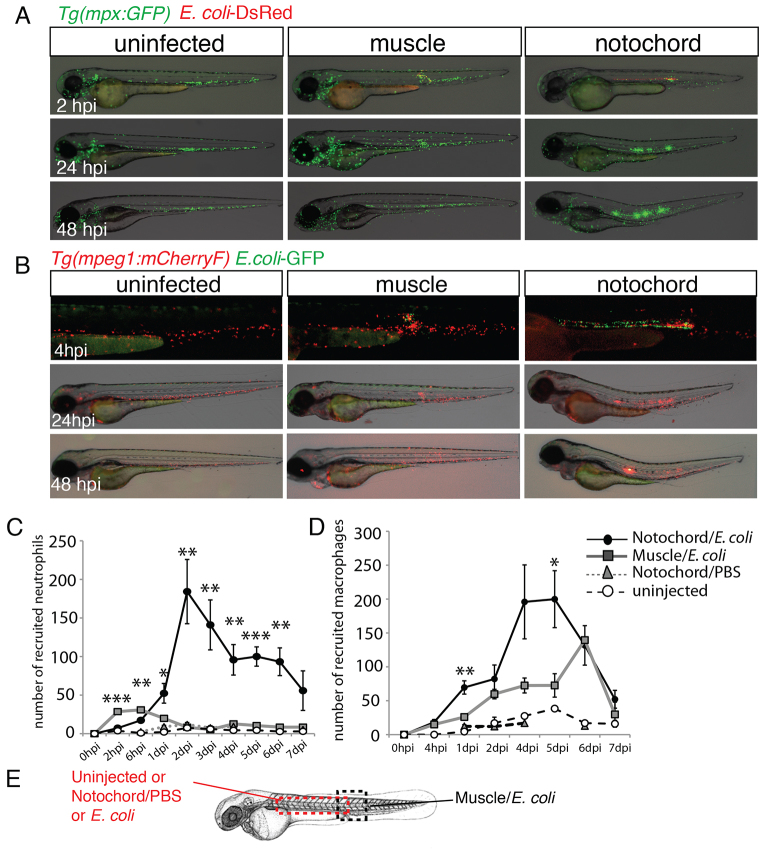
**Neutrophils and macrophages are recruited around the infected notochord.** (A) *Tg*(*mpx:GFP*) larvae were either left uninfected or infected with DsRed-expressing *E. coli* (red) in the muscle or notochord at 48 hpf. Neutrophil recruitment (green) was imaged repeatedly in individual larvae at the indicated timepoints. (B) A similar experiment to that shown in A was performed in *Tg*(*mpeg1:mCherryF*) larvae that were either uninfected or infected with GFP-expressing *E. coli* (green) in muscle or the notochord at 48 hpf. (C,D) Corresponding counts of neutrophils (C) and macrophages (D) over the course of 1 week post-infection. Results are expressed as the mean number of cells±s.e.m., five to nine fish were examined per timepoint, *P<0.05, **P<0.005 and ***P<0.001. (E) The dashed boxes in the diagram represent the zone where counting was performed.

We analysed the kinetics of immune cell recruitment using *Tg*(*mpx:GFP*) and *Tg*(*mpeg1:mCherryF*) lines by counting the leukocytes that accumulated around the infected notochord. As for muscle infections (Colucci-Guyon et al., 2011), notochord infections led to neutrophil recruitment within the very first hours following the injection. After injection of bacteria into the muscle, the number of neutrophils that had been recruited started decreasing at 24 hpi (Fig. 4A,C), whereas, in the notochord, neutrophil recruitment continued to increase at 48 hpi and remained high up to 6 days post-infection (dpi), forming one to four clusters that were towards the anterior of the injection site (20 out of 26 fish; Fig. 4A,C). Macrophages migrated to both infected muscle and notochord in a similar manner (Fig. 4B) – in both cases, the recruitment of macrophages lasted for 6 days, but more macrophages were recruited to the infected notochord (23 out of 23 fish; Fig. 4B,D). The observed immune response was not a consequence of the injection per se in the notochord, because the injection of PBS or fluogreen Dextran caused neither neutrophil nor macrophage recruitment (Fig. 4C,D; supplementary material Fig. S2).

We conclude that infection of the notochord induces a massive recruitment of macrophages and neutrophils to its periphery; remarkably, this inflammatory response is not properly resolved, as leukocyte accumulation persists for days, despite the clearance of bacteria in a matter of hours.

### Notochord inflammation induces notochord damage

**Fig. 5.**
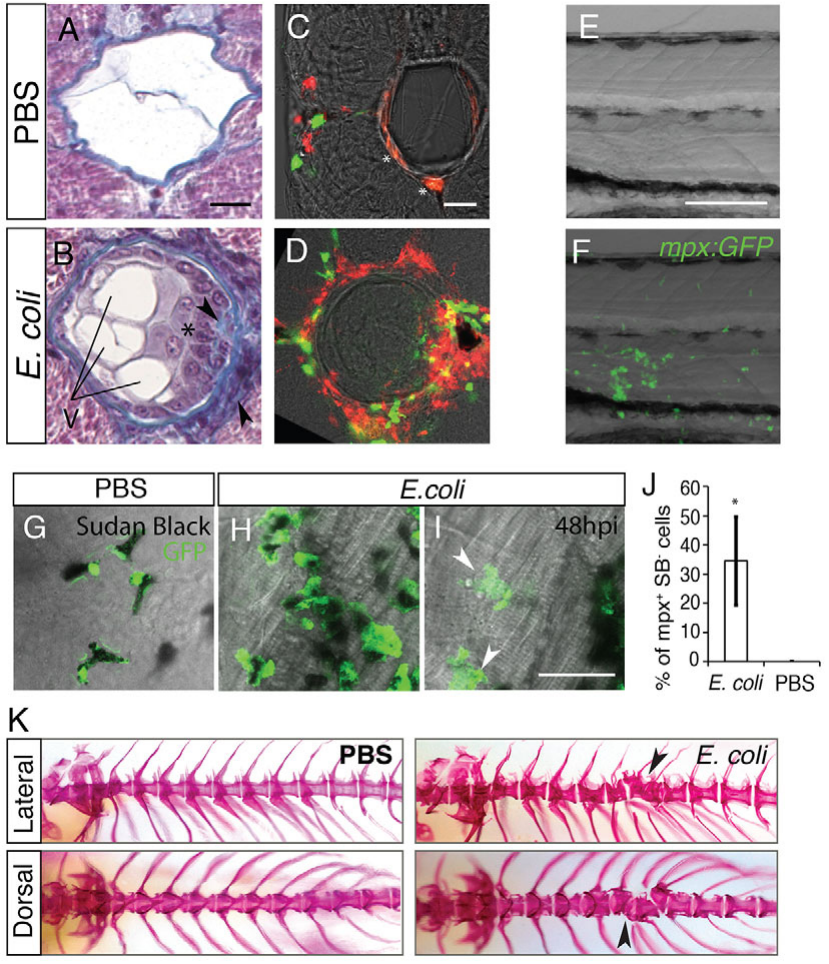
**Late consequences of notochord infection.** (A,B) Histological analysis at 5 dpi of larvae that had been injected in the notochord with PBS (A) or *E. coli* (B). Cross-sections (4 μm) stained with Masson’s Trichrome. In the infected larvae, collagen-enriched matrixes (blue) and supernumerary vacuoles (V) were seen. Cells inside the notochord (*) and ectopic matrix deposition (arrowhead) can be seen in the sections. (C,D) Neutrophil (green) and macrophage (red) infiltration in the notochord, multiscan confocal microscopy. Cross-sections (60 μm) of *Tg*(*mpx:GFP; mpeg1:mCherryF*) larvae at 5 days following injection of the notochord with PBS (C) or with *E. coli* (D). *Nonspecific staining. (E,F) Notochord of *Tg*(*mpx:GFP*) larvae at 3 dpi. (E) Transmitted-light image, overlaid in F with a maximal projection of confocal fluorescence images that show neutrophils (green) clustered at the damaged zone. (G–I) Sudan-black (SB) staining (black) and GFP expression (green) in *Tg*(*mpx:GFP*) larvae at 48 hours post-injection of either PBS (G) or *E. coli* (H,I) into the notochord. Arrowheads indicate neutrophils that have degranulated. (J) Graphic representation of the percentage of GFP^+^SB^−^ cells under the indicated conditions, mean±s.e.m., five or six fish per condition were analysed. *P<0.05 compared with the PBS control. (K) Infected larvae developed scoliosis and dysmorphic vertebrae. Larvae were injected with PBS or *E. coli* into the notochord at 48 hpf, grown to 60 dpf and fixed for analysis. Bones were stained with Alizarin red. Lateral and dorsal views of the anterior part of the vertebral column are shown. Arrowheads indicate mis-shaped and fused vertebral bodies. Scale bars: 20 μm (A-D,G-I); 50 μm (E,F).

Inflammation is fundamental in order to control bacterial infections. However, it is crucial that this inflammation resolves in a timely manner in order to prevent damage to the surrounding tissues. In order to analyse the consequences of notochord inflammation, we imaged larvae, in which bacteria had been injected into the notochord, for 10 days. At 5 dpi, most of the infected larvae presented curved malformation and lesions of the notochord, upstream of the injection site (43 out of 49 fish; supplementary material Fig. S3A,B). Although the defects were lessened, we still observed this malformation at 10 dpi (supplementary material Fig. S3C). Histological analysis of PBS-injected larvae at 5 dpi revealed normal architecture with highly vacuolated cells that were surrounded by a regular collagen sheath. By contrast, the infected larvae displayed swelling of the notochord at various locations along the antero-posterior axis (Fig. 5A,B; supplementary material Fig. S3D–K). At these spots, we observed an ectopic collagen-rich extracellular matrix, and an increased number of cells and vacuoles inside the notochord, suggesting the presence of supernumerary notochordal cells, rather than a phenomenon of fragmented vacuoles. Cells of a smaller size were also found clustered either outside or inside of the notochord; they were often trapped within the matrix, suggesting that they are infiltrating leukocytes (supplementary material Fig. S3J–K). Analysis by using confocal microscopy revealed that, although leukocytes were positioned at the margin of the myotome in PBS-injected larvae, they started infiltrating the notochord in *E. coli*-injected larvae from 2 dpi (Fig. 5C,D shows the 5 dpi timepoint; the data at 2 dpi is not shown), a timepoint that is long after bacterial clearance. Because notochord defects colocalised with neutrophil clusters (Fig. 5E,F), we asked whether neutrophil infiltration contributes to the destruction of host tissues. Indeed, this infiltration was concomitant with neutrophil degranulation – 35% of neutrophils in the inflammation region harboured no myeloperoxidase-containing granules, whereas all neutrophils in the control retained them (Fig. 5G–J). Taken together, these results show that infection of the notochord eventually leads to severe damage to the notochord tissue, which correlates with late leukocytic infiltration and neutrophil degranulation.

### Notochord inflammation triggers vertebral column defects

As the notochord participates to vertebral column formation we asked whether the inflammation of the notochord could trigger vertebral column defects. We thus examine the axial skeleton of juvenile fishes at 60 days post-fertilization (dpf) using Alizarin red staining. Zebrafish that had been previously injected with *E. coli* in the notochord developed scoliosis and dysmorphic vertebral column in their anterior region (upstream the injection site), characterised by fused and misshaped vertebral bodies and absence of intervertebral disc (11 out of 11 fish; Fig. 5K). No defects were observed in the PBS injected controls, excepting a single vertebral body that appeared more calcified at the injection site in few fishes (two out of 19 fish; Fig. 5K; data not shown).

### Neutrophils but not macrophages expressed high levels of il1b in notochord infected embryos

**Fig. 6.**
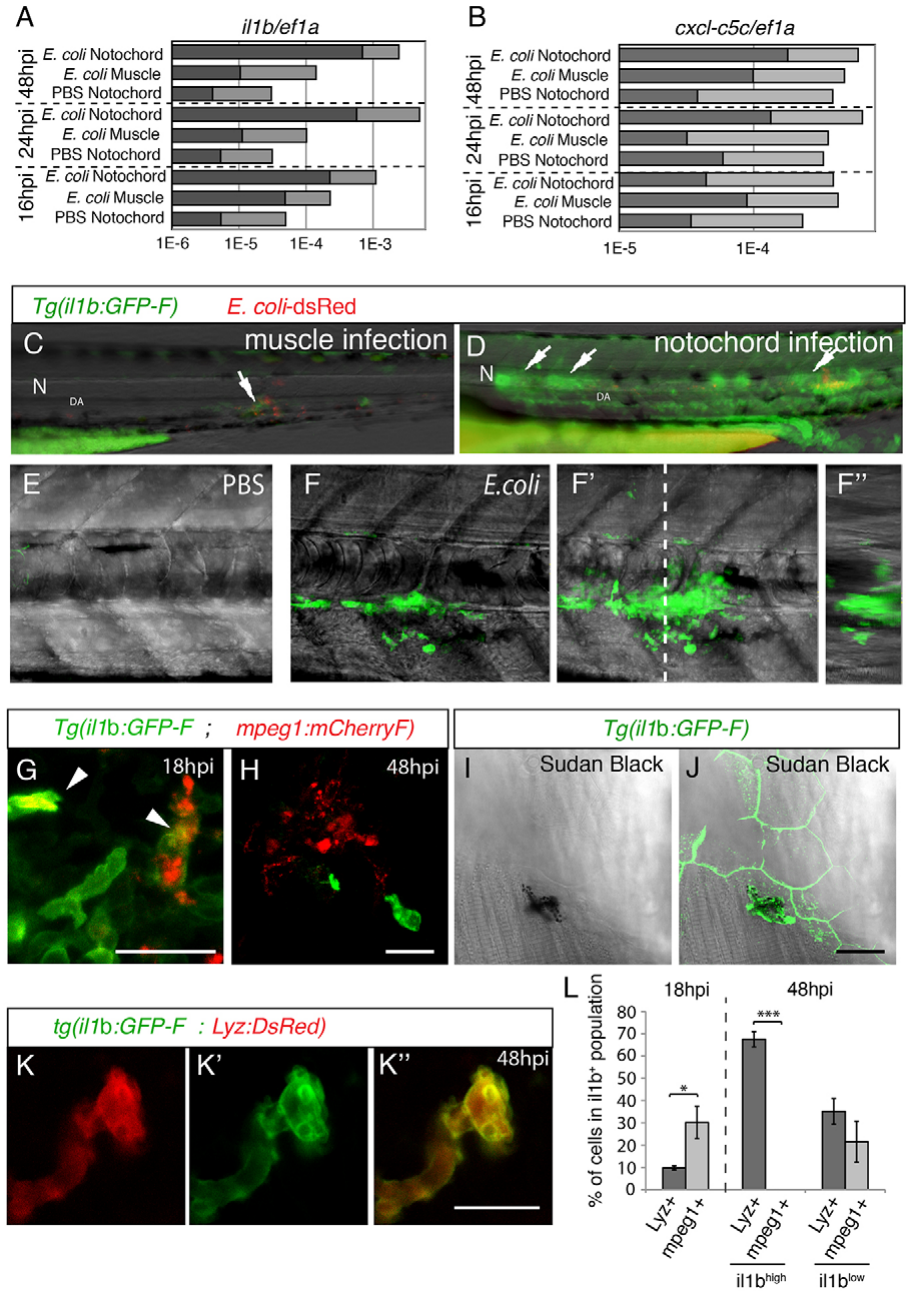
**GFP expression in *Tg*(*il1b:GFP-F*) infected larvae.** (A,B) The relative expression of *il1b* (A) and of *cxcl-c5c* (B) at different timepoints post-infection, under three different conditions – PBS injected into the notochord, *E. coli* injected into muscle and *E. coli* injected into the notochord. Quantitative real-time RT-PCR on whole embryo RNA extracts using *ef1a* as a reference gene was performed; light grey bars represent the 95% confidence intervals (Student’s *t*-test). Non-overlapping light grey bars indicate *P*<0.05. These results are representative of three independent experiments. (C,D) GFP expression at 24 hpi in *Tg*(*il1b:GFP-F*) larvae that had been injected with *E. coli* (red) into muscle (C) or the notochord (D). Arrows indicate expression next to inflammation sites. N, notochord; DA, dorsal aorta. (E,F) Multiscan confocal analysis of GFP expression in *Tg*(*il1b:GFP-F*) larvae in the notochord region 48 hours following the injection of PBS or *E. coli*. (E-F′) Maximum projections, (F) longitudinal- and (F″) cross-section views. The dashed line indicates the position of cross-section F″ relative to F. (G,H) The expression of mCherry (red) and GFP (green) in *Tg*(*mpeg1:mCherryF ; il1b:GFP-F*) double transgenic larvae at 18 (G) and 48 hpi (H) in the notochord region was analysed by using confocal microscopy. Arrowheads indicate macrophages expressing GFP. (I,J) Sudan-black staining and GFP expression in *Tg*(*il1b:GFP-F*) larvae was analysed by using confocal microscopy at 48 hpi. The images show a GFP^+^ neutrophil underneath GFP^+^ keratinocytes. (K-K″) The expression of DsRed (red) and GFP (green) in *Tg*(*Lyz:DsRed ; il1b:GFP-F*) double transgenic larvae at 48 hpi was analysed by using confocal microscopy. A merged image is shown on the right. (L) Quantification of *il1b*^+^; *mpeg1*^+^ and *il1b*^+^; *Lyz*^+^ cells in the inflammation region. Counts are expressed as the percentage of cells within the population expressing GFP (*il1b*^+^, *il1b*^low^ or *il1b*^high^). *P<0.05, **P<0.005 and ***P<0.001. Scale bars: 20 μm.

In order to decipher the molecular mechanisms that orchestrate the inflammatory response to notochord infection, we analyzed the expression of many cytokines and chemokines in larvae that had been infected in the notochord (supplementary material Table S1). The expression of most of the chemokines that we examined was unchanged, such as that of the chemokine Cxcl-c5c; however, transcripts of the proinflammatory cytokine Il1b were strongly upregulated from 1 dpi onwards (Fig. 6A,B).

To study the spatiotemporal behaviour of the cells that produce the *il1b* transcripts, we established a transgenic line that expressed farnesylated GFP (membrane-targeted GFP; GFP-F) under the control of the *il1b* promoter *Tg*(*il1b:GFP-F*). Uninfected *Tg*(*il1b:GFP-F*) developing embryos expressed GFP-F during the segmentation period in the elongating tail bud (supplementary material Fig S4). At 24 hpf, GFP expression appeared in tail keratinocytes, the olfactory epithelium and individual cells on the yolk sac that behave as primitive leukocytes. At 35 hpf, when leukocytes appear in the caudal hematopoietic tissue (CHT), scattered GFP^+^ cells were also found in the CHT region. From 50 hpf, GFP was expressed in keratinocytes at the tip of the caudal fin, in fin buds, retina, neuromasts, gills and thymus (supplementary material Fig. S4; data not shown). Whole-mount *in situ* hybridisation analysis confirmed the expression of endogenous *il1b* mRNA in the different organs that are cited above, showing that *Tg*(*il1b:GFP-F*) recapitulates *il1b* transcriptional expression (supplementary material Fig. S5A,C). Furthermore, upon stimulation by ‘danger signals’, both the neutrophils and macrophages of *Tg*(*il1b:GFP-F*) larvae strongly expressed GFP, correlating with the upregulation of the endogenous *il1b* mRNA that was observed in both leukocyte populations (supplementary material Fig. S6).

To study *il1b* transcription upon infection, we injected fluorescent bacteria (*E. coli*-DsRed) into the notochord of *Tg*(*il1b:GFP-F*) embryos. Specific expression of GFP was detected along the notochord as early as 18 hpi, which increased significantly by 48 hpi and was maintained up to 5 to 6 dpi before decreasing (24 out of 36 fish; Fig. 6D; supplementary material Fig. S5D). The induction of GFP upon infection correlated with the upregulation of *il1b* mRNA (supplementary material Fig. S5A–C). Conversely, control PBS and muscle injections, respectively, induced no and low expression of GFP around the injection site at 24 hpi and 48 hpi (Fig. 6C,E; supplementary material Fig. S5D). The analysis of larvae that had been injected in the notochord revealed that GFP was expressed by leukocytes that were clustered around the notochord but not by notochordal cells (Fig. 6F). Additional GFP expression was detected in leukocytes that were scattered throughout the body and in the keratinocytes surrounding the inflammation site (supplementary material Fig. S5E,F; Fig. 6J).

By early timepoints (18 hpi) 30% of GFP-positive cells (*il1b^+^*) also expressed the macrophage marker *mpeg1* (*mpeg1^+^*), whereas only 10% expressed the neutrophil marker *lysosymeC* (*lyz^+^*) (Fig. 6G,L). During the late stages of inflammation (48 hpi), two distinct populations of GFP-expressing cells were observed around the inflamed notochord – cells that expressed a high level of GFP [*il1b*^high^, 60±6.5% (mean±s.e.m.)] and those that expressed a low level of GFP (*il1b*^low^, 40±6.5%). At 48 hpi, no macrophages were *il1b*^high^, whereas neutrophils (*lyz^+^*) comprised 68% of these *il1b*^high^ cells. The other 32% of *il1b*^high^ cells were *mpeg1^−^* and *Lyz*^−^ and unidentifiable. The remaining *il1b*^low^ cell population comprised 35% neutrophils (*il1b*^low^ *Lyz^+^*) and 27% macrophages (*il1b*^low^ *mpeg1^+^*) (Fig. 6H–L).

In conclusion, our data suggest that Il1b production dynamically shifts from early timepoints of the inflammatory response – at which both macrophages and neutrophils contribute to Il1b production – to late phases, when neutrophils are the main source of Il1b.

### Il1b is required for the early and late recruitment of neutrophils but not macrophages

**Fig. 7.**
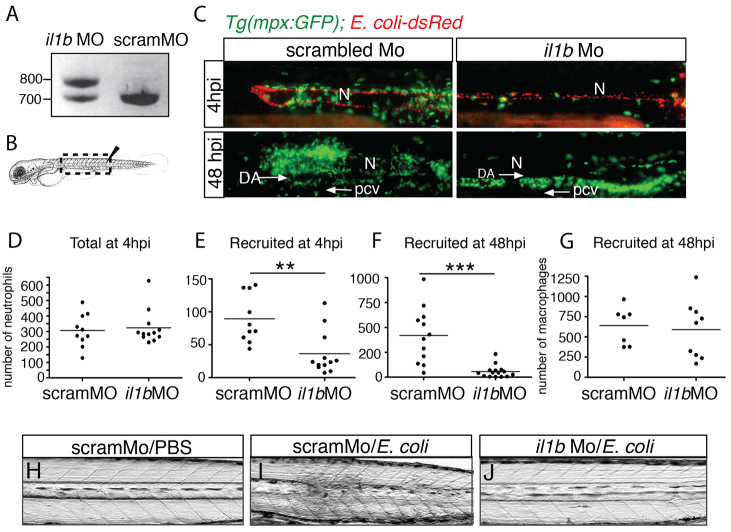
**Il1b is required for the early and late recruitment of neutrophils but not macrophages.** PCR amplification of *il1b* cDNA from the 48 hpf intravenous-infected larvae, which had previously been injected with either scambled morpholino (scramMO) or with a morpholino antisense oligonucleotide that blocked the splicing of intron 2 (*il1b*MO). (B) The diagram shows the region that is imaged in C (dashed box). (C) Recruitment of neutrophils (green) in *Tg*(*mpx:GFP*) larvae at 4 hours and 48 hours following the injection of DsRed-expressing *E. coli* (red) into the notochord. These larvae had been previously injected with scrambled morpholinos (scramMO) or *il1b* MO. N, notochord. At 48 hpi, many neutrophils were found in the AGM region, in between the dorsal aorta (DA) and the posterior caudal vein (pcv); arrows indicate the direction of blood flow. (D–G) Plots show the number of total neutrophils at 4 hpi (D) and the number of recruited neutrophils at 4 hpi (E) and 48 hpi (F). (G) The number of recruited macrophages at 48 hpi in similar experiments that were performed using *Tg*(*mpeg1:mCherryF*) larvae. Each point represents an individual larva, the bar represents the mean value. **P<0.005, ***P<0.001. (H-J) Bright-field images of control (scramMO) and *il1b* morphants 3 days after injection of either PBS or *E. coli* into the notochord. Lateral views of the trunk region are shown in C,H,I,J.

Morpholino-mediated gene knockdown was used to investigate the role of zebrafish Il1b during severe notochord inflammation. Reverse transcription (RT)-PCR analysis, performed using primers on both sides of the splice site that is targeted by the morpholino, provided evidence of strong blocking of splicing, whereas overall morphology was not noticeably affected (Fig. 7A; the general morphology of the morphants is not shown). The *il1b* morpholino was injected into *Tg*(*mpx:GFP ; mpeg1:mCherryF*) embryos, and the morphants were infected by the injection of *E. coli* into the notochord. In embryos that had not been injected with morpholino, or those that had been injected with control scrambled morpholino, both neutrophils and macrophages were recruited to the inflamed notochord in a normal manner. In *il1b* morphants, neutrophil recruitment was slightly reduced at 4 hpi and strongly reduced at 48 hpi compared with controls (Fig. 7C,E–G); only macrophages localised around the notochord of *il1b* morphants at 4 and 48 hpi (Fig. 7G; supplementary material Fig. S7). The decreased neutrophil recruitment was not caused by a general reduction of the neutrophil population under steady-state conditions because the total number of neutrophils in *il1b* morphants was similar to that in uninjected embryos or embryos that had been injected with the control scrambled morpholino at 4 hpi (Fig. 7D for *il1b* morpholino compared to scrambled morpholino; data not shown for *il1b* morphants compared to uninjected larvae). Additionally, a reduction of the overall inflammatory response was excluded because macrophage recruitment was unaffected (Fig. 7G). Long-term examination of the infected *il1b* morphants revealed that loss of *il1b* prevented formation of the lesions that were often detected in the rostral part of the notochord after infection (Fig. 7H–J, lesions were found in one out of 12 *il1b* morphant fish and seven out of 11 scrambled morpholino fish). Taken together, these results establish an important and specific role for Il1b in the recruitment of neutrophils during chronic inflammation of the notochord and subsequent sequelae.

## DISCUSSION

This article describes a model of zebrafish notochord infection with *E. coli* that recapitulates several of the inflammatory aspects of bone and cartilage infection in human. We thus propose that the specific advantages of the larval zebrafish model system – transparency, genetic tractability – should facilitate the study of the early cellular and molecular inflammatory mechanisms that are related to matrix-tough tissue infections.

### Notochord infection of the zebrafish larvae as a model to study inflammatory aspects of bone and cartilage infections

The similarity of the notochord with cartilage (in terms of transcriptional profile) and vertebral bone (in terms of structure and function) (Fleming et al., 2001; Stemple, 2005) prompted us to establish a model of the infection of this organ in order to mimic the inflammatory aspects of human osteomyelitis and osteoarthritis. As in some infections of human bones and joints, infection of the zebrafish notochord induces a severe inflammation that greatly outlasts the presence of live microbes and thus can be considered ‘chronic’ – taking into account due allowances. Indeed, injection of *E. coli* into the notochord leads to the rapid recruitment of both neutrophils and macrophages to the infected notochord within the first hour. The injection needle locally disrupts the collagen sheath, creating an opening for the leukocytes to phagocytose some bacteria in the notochord. However, probably because of the turgidity of the notochordal cells within the thick collagen sheath, neutrophils and macrophages fail to enter the notochord at sites distant from the injection site. This finding suggests that, in contrast to what is observed for transendothelial migration, leukocytes do not easily pass through all matrixes. Even though the injected bacteria are cleared in less than one day, neutrophil and macrophage recruitment along the notochord persists for days, preventing the resolution of inflammation. Although they, initially, randomly spread around the notochord at 2 to 24 hpi, leukocytes accumulate in patches in the later stages of the response (from 48 hpi), suggesting that they are involved in the activation and recruitment of more leukocytes. Granules, the hallmarks of granulocytes, are stores of enzymes that participate in killing microbes and digesting tissues. We found that, from 2 dpi, despite the fact that injected microbes have been cleared by this point, neutrophils degranulate next to the notochord. Thus, the release and activation of proteolytic enzymes that are able to disassemble the collagen fibrils that impede neutrophil-bacteria contacts might explain the infiltration of neutrophils into the notochord, which is observed in the late phases of inflammation (Nathan, 2006). This neutrophil degranulation might be responsible for the tissue damage that is observed in notochord-infected larvae – including perturbation of extracellular matrix deposition, swelling and curvature of the notochord, and cellular clusters within the notochord that are reminiscent of the abscesses observed in human osteomyelitis (Lew and Waldvogel, 2004; Wright and Nair, 2010). The notochord lesion, which is induced by the severe inflammatory episode might contribute to the dismorphic vertebrae that were observed during adulthood. Although extracellular matrix deposition, neutrophil infiltration and tissue damage are the hallmarks of chronic infection, which occur in some osteomyelitis and septic arthritis individuals, curvature of the axial skeleton is reminiscent of that of observed in individuals that have bone tuberculosis. Indeed, also known as Pott disease, which accounts for 1–2% of overall tuberculosis cases, it is usually characterised by the spread of tuberculosis bacilli into the disc space from the vascular system, resulting in the destruction of the disc and progression to the bone of adjoining vertebral bodies. As the vertebrae degenerate and collapse, a kyphotic deformity results (Moon, 1997). The chronic inflammation of the zebrafish notochord after bacterial clearance described here is also reminiscent of autoinflammatory disorders – such as chronic non-bacterial osteomyelitis, deficiency of IL-1β receptor antagonist or neonatal-onset multisystem inflammatory disease. Although the etiology of chronic non-bacterial osteomyelitis is still unknown, deficiency of IL-1β receptor antagonist and neonatal-onset multisystem inflammatory disease are caused by mutations in the *IL1RN* and *NLRP3* genes, respectively, and result in over-activation of the IL-1β pathway (Morbach et al., 2013). Accordingly, neutrophil recruitment to the infected zebrafish notochord was found to be dependent on Il1b.

### Elimination mechanism of *E. coli* in the notochord

Many bacteria avoid the immune response by hiding inside of cells of their host. By doing so, they are out of reach of the immune system and multiply within these cells before further invasion of the body. We have previously shown that, when injected into the notochord, non-pathogenic *Mycobacterium marinum Tes:A* mutant bacteria are internalised by notochordal cells as revealed, using TEM, by the presence of the bacteria in endosomes (Alibaud et al., 2011). Interestingly, similar bacterial internalisation has previously been reported for osteoblasts and chondrocytes (Diaz-Rodriguez et al., 2009; Wright and Nair, 2010; Jiao et al., 2013, **Castillo and Kourí, 2004). All of these observations suggest that osteoblasts, chondrocytes and notochordal cells are non-professional phagocytes that engulf invader microbes. However, their phagocytic ability depends on the nature of these microbes. We observe here that notochordal cells do not phagocytose *E. coli*; bacteria remain extracellular for a few hours before being eliminated, as demonstrated by CFU assays, and fluorescence and electron microscopy. How are they killed? As leukocytes cannot phagocytose them, another mechanism must be used. The ‘unhealthy’ shape of some *E. coli* within the notochord, as observed by electron microscopy, suggests death by a humoral lysis mechanism that remains to be identified. The complement system is an obvious candidate, and we are currently studying the contribution of complement genes to the observed bacterial clearance.

### Putative functions of Il1b in zebrafish

IL-1β is a critical pro-inflammatory mediator of the host response to microbial infection. It acts on almost all cell types and has many functions – including growth and proliferation, the induction of adhesion molecules in epithelial cells, the activation of phagocytosis and release of proteases in macrophages and neutrophils, and the activation of B and T cells (Dinarello, 2009). Using transgenic zebrafish larvae that expressed farnesylated GFP under the control of the *il1b* promoter, we show here, for the first time, the dynamic transcriptional activity of *il1b* in a whole organism. Its expression during zebrafish development, especially in the keratinocytes of the caudal and pectoral fin bud, correlates with the regions of the body where active keratinocyte proliferation occurs. This is in agreement with findings in mammals where keratinocytes express IL-1β (Kupper et al., 1986). However, resting keratinocytes express IL-1β in its unprocessed biologically inactive form – pro-IL-1β. Indeed, to be secreted as an active cytokine, pro-IL-1β must be cleaved by caspases (caspase-1, caspase-11 and others), which are themselves activated through inflammasome assembly (Kayagaki et al., 2011). Although *il1b* transcription is low in most cell types and strongly inducible following the detection of microbial molecules – notably in myeloid cells – constitutive expression of pro-IL-1β by some tissues should allow for a faster response to external injuries. This is logical for cells that are directly exposed to the environment – such as keratinocytes, olfactory epithelial cells, enterocytes or neuromast cells – all cell types that were seen to spontaneously express endogenous *il1b* mRNA and GFP in the Tg(*il1b:GFP-F*) line. In zebrafish, Il1b has been shown to be structurally conserved compared with its human counterpart, and the Il1b pathway is also dependent on caspases (Ogryzko et al., 2014). Whether the active form of Il1b is produced in these zebrafish organs remains to be determined. Notably, spontaneous GFP expression in some cell types, such as those of neuromasts and the gut, mimics the spontaneous expression of GFP in the *Tg*(*NFκB:EGFP*) reporter transgenic zebrafish line (Kanther and Rawls, 2010), which is in agreement with the well-known contribution of NF-κB to *il1b* induction (Ogryzko et al., 2014).

In addition to its basal expression during development, GFP is also induced following *E. coli* infection in *Tg*(*il1b:GFP-F*) fish, which parallels endogenous *il1b* induction. This induction is strictly correlated in intensity and time with the recruitment of leukocytes. Indeed, infection in muscles induces a low level of expression that quickly resolves, whereas infection of the notochord induces strong and persistent expression. The GFP expression level decreases one day before the resolution of inflammation. In most mammalian systems, IL-1β has been reported to be mainly produced by circulating monocytes, tissue macrophages and dendritic cells (Dinarello, 2009). Here, we describe that, in our zebrafish notochord infection model, *il1b* is first expressed in macrophages during the early phase of inflammation and is mainly expressed by neutrophils during the late phases. This is reminiscent of the recent work of Karmakar and collaborators, which shows that neutrophils are the main source of Il1b in response to *Pseudomonas aeruginosa* in mouse, highlighting the importance of activated neutrophils in mediating inflammation (Karmakar et al., 2012).

One interesting question arising from the present work is which signal stimulates the recruitment of leukocytes to the infected notochord? Is it a primary signal that is actively emitted by the notochordal cells, does it come from the injected bacteria or does it correspond to bacterial leftovers after destruction within the notochord? We are currently investigating this latter hypothesis through the injection of different bacterial products into the notochord of larvae and evaluating their ability to recruit leukocytes.

Our data demonstrate that *il1b* is not expressed by notochordal cells after *E. coli* injection; however, it is partially required for the initial recruitment of neutrophils, even though a low number of neutrophils is still recruited to the infected notochord in the absence of *il1b. Il1b* is also required for the persistence of neutrophils at the inflammation site, but it has no effect on macrophage recruitment, suggesting that Il1b is a signal produced by recruited leukocytes themselves that amplifies the activation of neutrophils and prevents resolution of inflammation. If neutrophils do not encounter a bacterium, they are thought to degranulate to promote tissue digestion in order to gain access to bacteria (Nathan, 1987). Indeed, we show that the notochord is not directly accessible to neutrophils, thus degranulation might produce cellular and matrix debris that maintain inflammation around the notochord. Therefore, multiple different signals probably induce and maintain the inflammatory response.

IL-1β is a key player in the inflammatory process that occurs in bone diseases (Wright and Nair, 2010). The neutralisation of IL-1β signalling appears to be crucial for the potential therapy of osteomyelitis, but also for other inflammatory diseases, such as rheumatoid arthritis. Several specific drugs that inhibit this pathway are currently used to improve the symptoms of these diseases (Dinarello, 2004). Interestingly, in our model, knocking down *il1b* suppressed the long lasting inflammation. We therefore consider the zebrafish model to be perfectly adapted to the challenge of dissecting the exact function of *il1b* during chronic inflammation of bone and joints.

## MATERIALS AND METHODS

### Ethics statement

All animal experiments that are described in the present study were conducted at the University Montpellier 2 according to European Union guidelines for the handling of laboratory animals (http://ec.europa.eu/environment/chemicals/lab_animals/home_en.htm) and were approved by the Direction Sanitaire et Vétérinaire de l’Hérault and Comité d’Ethique pour l’Expérimentation Animale under reference CEEA-LR-13007.

### Zebrafish line and maintenance

Fish maintenance, staging and husbandry were as described previously (Alibaud et al., 2011) under standard conditions (Westerfield, 2007). Experiments were performed using the F1 golden zebrafish mutant (Lamason et al., 2005), originally purchased from Antinea, and using the transgenic line *Tg*(*mpx:GFP*) (kind gift from Steve Renshaw, MRC Centre for Developmental and Biomedical Genetics, Sheffield, UK) to visualise neutrophils. Embryos were obtained from pairs of adult fish by natural spawning and raised at 28.5°C in tank water. Embryos and larvae were staged as described previously (Kimmel et al., 1995).

### Statistical analysis

Student’s *t*-tests (two-tailed, unpaired) were performed in Fig. 1K; Fig. 4C,D; Fig. 5J; Fig. 6L and Fig. 7D–G.

For statistics on the survival of larvae that had been infected with *E. coli*, the survival rate of each of the populations of injected embryos was compared with that of the uninjected embryos using the log-rank (Mantel–Cox) test using GraphPad Prism 4 (Graphpad Software).

### Transgenic line construction

Based upon the work of Ellet et al. (Ellett et al., 2011), we cloned a new version of the *mpeg1* promoter to drive the specific expression of a red fluorescent membrane-targeted protein. A 1.9-kb fragment of the *mpeg1* promoter was amplified from genomic DNA using primers zMpeg1P4 (5′-TTGGAGCACATCTGAC-3′) and zMpeg1E2N2 (5′-TTATAGCGGCCG -CGAAATGCTCTTGACTTCATGA-3′), digested by NotI and then ligated to the reading frame of the farnesylated mCherry (mCherry-F) protein so that the *mpeg1* AUG was in-frame with the downstream mCherry-F open reading frame. The plasmid was injected in one-cell stage embryos together with the Tol2 transposase RNA. F0-microinjected embryos were screened using the expression of the transgene in macrophages, as described previously (Ellett et al., 2011), and stable lines were established. The same strategy was used to clone a 3.4-kb fragment of the *il1b* promoter using primers P2 (5′-ATGAGCTCCGAAATTCAGCTGG-3′) and E2N (5′-TTATAGCGGCCGCTTGCCCGCATGCCATCATTTCTA-3′).

### Quantitative real-time RT-PCR analysis of cytokines

To determine the relative expression of *il1b* and *cxcl-c5c*, total RNA from infected larvae and controls (pools of ~10 larvae each) was prepared at different timepoints post-infection. RNA preparation, reverse transcription and quantitative real-time PCR, including statistical analysis, have been described elsewhere (Aggad et al., 2009). The primers used were as follows: zIL1b.fw, 5′-TGGACTTCGCAGCACAAAATG-3′; zIL1b.rv, 5′-GTTC -ACTTCACGCTCTTGGATG-3′; zEF1a.fw, 5′-TTCTGTTACCTGGCAAAGGG-3′; zEF1a.rv, 5′-TTCAGTTTGTCCAACACCCA-3′; CXCL-C5C.fw, 5′-AACCAAGTCCATCCTGCTCA-3′; CXCL-C5C.rv, 5′-CGTTAGGATCCAAACACACCT-3′.

### Embryo and larva manipulation

For *il1b* loss-of-function assays, we designed morpholino antisense oligonucleotides that specifically hybridised with the splicing site between intron 2 and exon 3 – morpholino sequence 5′-CCCACAAACTGCAAAATATCAGCTT-3′. The oligonucleotides against *il1b* (3 nl at a final concentration of 0.5 mM), or scrambled morpholinos (5′-AATCACAAGCAGTGCAAGCATGATG-3′, 3 nl at a final concentration of 0.5 mM) were injected into one-cell stage embryos. No side effect was observed. Efficiency was tested by using RT-PCR with primers for both sides of the morpholino target sequence: zil1b.30, 5′-TGCCGGTCTCCTTCCTGA-3′ and zil1b.50, 5′-GCAGAGGAACTTAACCAGCT-3′ (Fig. 7A). Sequencing confirmed the retention of the second intron of *il1b*, leading to a frame shift and premature stop codon. Larvae were infected with *Escherichia coli* K12 bacteria that harboured DsRed (van der Sar et al., 2003), Crimson (Clontech), or a GFP expression plasmid and had been grown in Luria broth (LB) with appropriate antibiotics. Overnight stationary-phase cultures were centrifuged (1 minute, 10,000 ***g***), and the bacteria were resuspended in sterile PBS (2×10^9^–4×10^9^ CFU/ml) and then injected into the muscle, caudal vein or notochord of 48- to 50-hpf larvae (Alibaud et al., 2011; Colucci-Guyon et al., 2011).

### Imaging of live zebrafish larvae

Larvae were anaesthetised in 0.01% 3-amino benzoic acidethylester (Tricaine), positioned on 35-mm glass-bottomed dishes (WillCo-dish^®^), immobilised in 0.8% low-melting-point agarose and covered with 2 ml of embryo water containing Tricaine. Epifluorescence microscopy was performed by using an MVX10 Olympus MacroView microscope that was equipped with MVPLAPO 1× objective and XC50 camera. Confocal microscopy was performed using a Leica SPE upright microscope with 40× HCX APO L 0.80 W and 20× CHX APO L 0.5 W objectives. Time-lapse acquisitions were performed at 23–26°C. The 4D files generated by the time-lapse acquisitions were processed using ImageJ. Three-dimensional reconstructions and optical sectioning were performed on the four-dimension files using Imaris (Bitplan AG, Zurich, Switzerland).

### CFU count

Individual larvae were lysed in 1% Triton-X100 and homogenised with a 26G syringe five to six times. The resultant debris and notochords were washed twice in PBS and incubated for 30 minutes at 30°C with agitation in the presence of 10 mg/ml collagenase that had been dissolved in PBS supplemented with 1 mM CaCl_2_. A fraction of the total lysate was plated on selective LB agar plates.

### Histology and sectioning

Larvae were fixed at 4-5 days post-infection in Bouin’s solution and washed in 70% ethanol. After successive dehydration, butanol and Paraplast baths, larvae were embedded in Paraplast X-TRA (Sigma), sectioned (using a Leitz Wetzlar Microtome), stained with Masson’s trichrome and imaged (using a Nanozoomer Slide scanner with 40× objective). For bone staining, juvenile fish were fixed in 4% paraformaldehyde in PBS for 48 hours at 4°C, dehydrated with 100% ethanol over 4 days, stained with Alizarin red (Javidan and Schilling, 2004) and transferred to 100% glycerol over the course of 1 week before analysis and storage.

### Isolation of mRNA from fluorescently labelled neutrophils and macrophages

Double transgenic larvae *Tg*(*mpx:eGFP/mpeg1:mCherry-F*) were injected with *E. coli* K12 or PBS into the notochord at 2 dpf. At 2 dpi, 40 anaesthetised larvae per sample were allocated to each well of a 6-well plate and dissociated in 2 ml of trypsin 0.25% with 0.5 mM EDTA for 1 hour at 33°C with pipetting every 10 minutes for correct dissociation. Reactions were stopped by the addition of CaCl_2_ (final concentration 1 mM) and foetal calf serum (final concentration 10%). The lysates were passed through a 40-μm mesh filter, washed twice with 1% FCS in PBS and sorted by fluorescence-activated cell sorting (MoFlo Astrios EQ) for GFP-labelled neutrophils and mCherry-labelled macrophages for collection directly into lysate buffer solutions for mRNA extractions using the RNAqueous-micro kit (Ambion).

### Larva dissociation and cell culture

Double transgenic larvae *Tg*(*il1b:eGFP/mpeg1:mCherry-F*) or *Tg*(*il1b:eGFP/LysC:mCherry*) at 4 dpf were anaesthetised and trypsinised individually in 6-well plates, as described above. After washing with 1% FCS in PBS, the cell suspensions were cultured in Leibovitz L-15 medium (21083-027, Gibco) for 1 hour at 30°C. Non-attached cells were then removed, and the attached macrophages and neutrophils were supplied with fresh L-15 medium and incubated at 30°C for an additional 3 hours before confocal imaging.

## Supplementary Material

Supplementary Material
