# Supplementary material for: Transient infection of the zebrafish notochord with E. coli induces chronic inflammation
Source: Dis Model Mech. 2014 Jul;7(7):871–82. doi: 10.1242/dmm.014498 (PMC4073276; doi:10.1242/dmm.014498)
Supplement: Supplementary Material [file supp_7_7_871__index.html]

Transient infection of the zebrafish notochord with E. coli induces chronic inflammation — Supplementary Material 

# Transient infection of the zebrafish notochord with *E. coli* induces chronic inflammation

## DMM014498 Supplementary Material

**Files in this Data Supplement:**

- **Supplementary Material**
