# Supplementary material for: Transient infection of the zebrafish notochord with E. coli induces chronic inflammation
Source: Dis Model Mech. 2014 Jul;7(7):871–82. doi: 10.1242/dmm.014498 (PMC4073276; doi:10.1242/dmm.014498)
Supplement: Supplementary Material [file supp_7.7.871_DMM014498.pdf]

SUPPLEMENTARY FIGURES AND TABLES AND THEIR LEGENDS

Transient infection of the zebrafish notochord triggers chronic inflammation

Mai Nguyen-Chi [1,2,5], Quang Tien Phan [1,2,5], Catherine Gonzalez [1,2], Jean-François Dubremetz [1,2], Jean-Pierre Levraud [3,4] and Georges Lutfalla [1,2].

[1]Dynamique des Interactions Membranaires Normales et Pathologiques, Université Montpellier 2, UMR 5235, case 107, Place Eugène Bataillon, 34095 Montpellier Cedex 05, France.

[2]CNRS; UMR 5235, Place Eugène Bataillon, 34095 Montpellier Cedex 05, France.

[3]Macrophages et Développement de l'Immunité, Institut Pasteur, Paris F-75015, France.

[4]CNRS URA2578, Paris F-75015, France

[5]These authors contributed equally to this work

Correspondence to Mai Nguyen Chi and Georges Lutfalla, Dynamique des Interactions Membranaires Normales et Pathologiques, Université de Montpellier 2, UMR 5235, CNRS, case 107, Place Eugène Bataillon, 34095 Montpellier Cedex 05, France.

E-mail addresses: mai.nguyen-chi@univ-montp2.fr and lutfalla@univ-montp2.fr

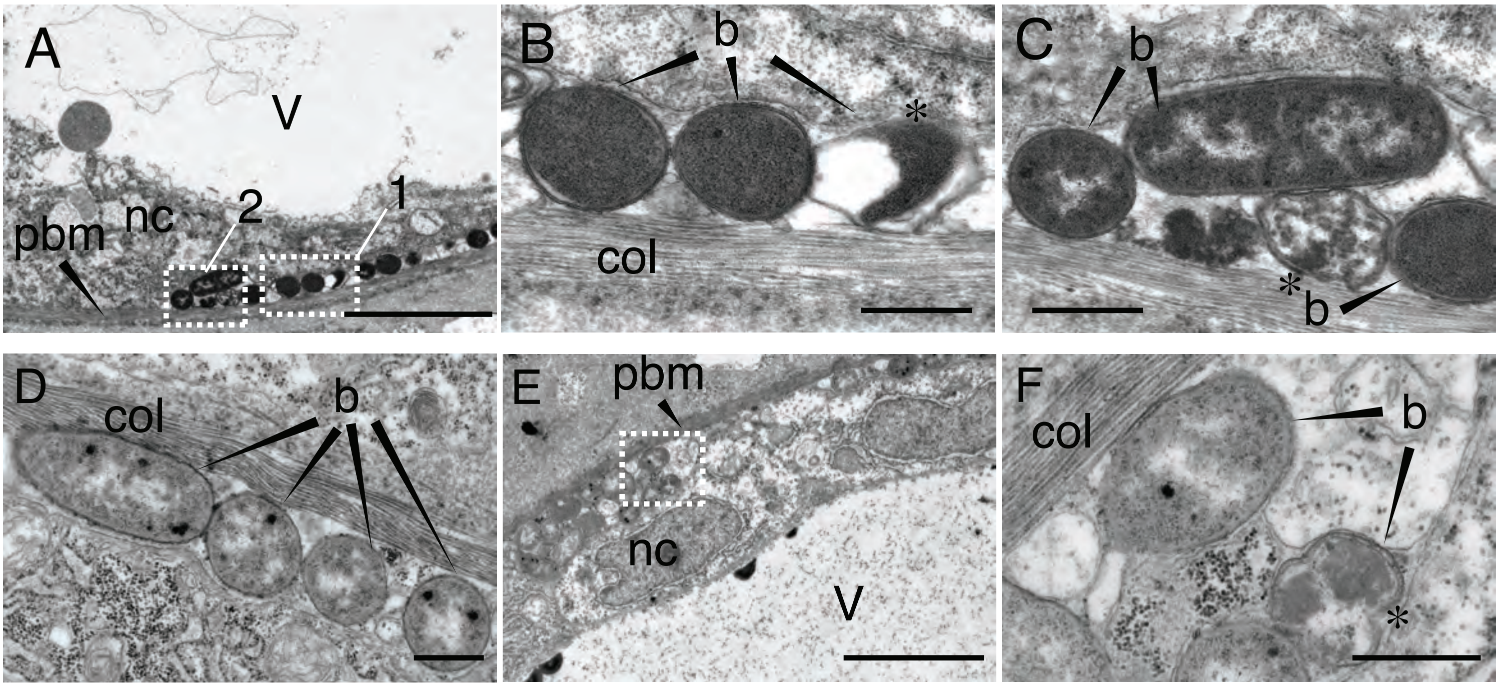

Figure S1: ***E. coli*** **remains extracellular in the notochord of infected larvae.**
Ultra-structural analysis using transmission electron microscopy of notochord infected larvae with *E. coli*, 15 min after infection (A-C) and 2 hpi (D-F). (B) and (C) are high magnification of regions boxed in (A), box 1 and 2, respectively. and (F) of region boxed in (E). Asterisks show bacteria whose morphology is altered. b: bacteria, pbm: peri-notochordal basement membrane, col: collagen sheath, nc: notochordal cell, v: vacuole. Scale bars : 5 µm in A and E, 0.5 µm in B, C, D and F.

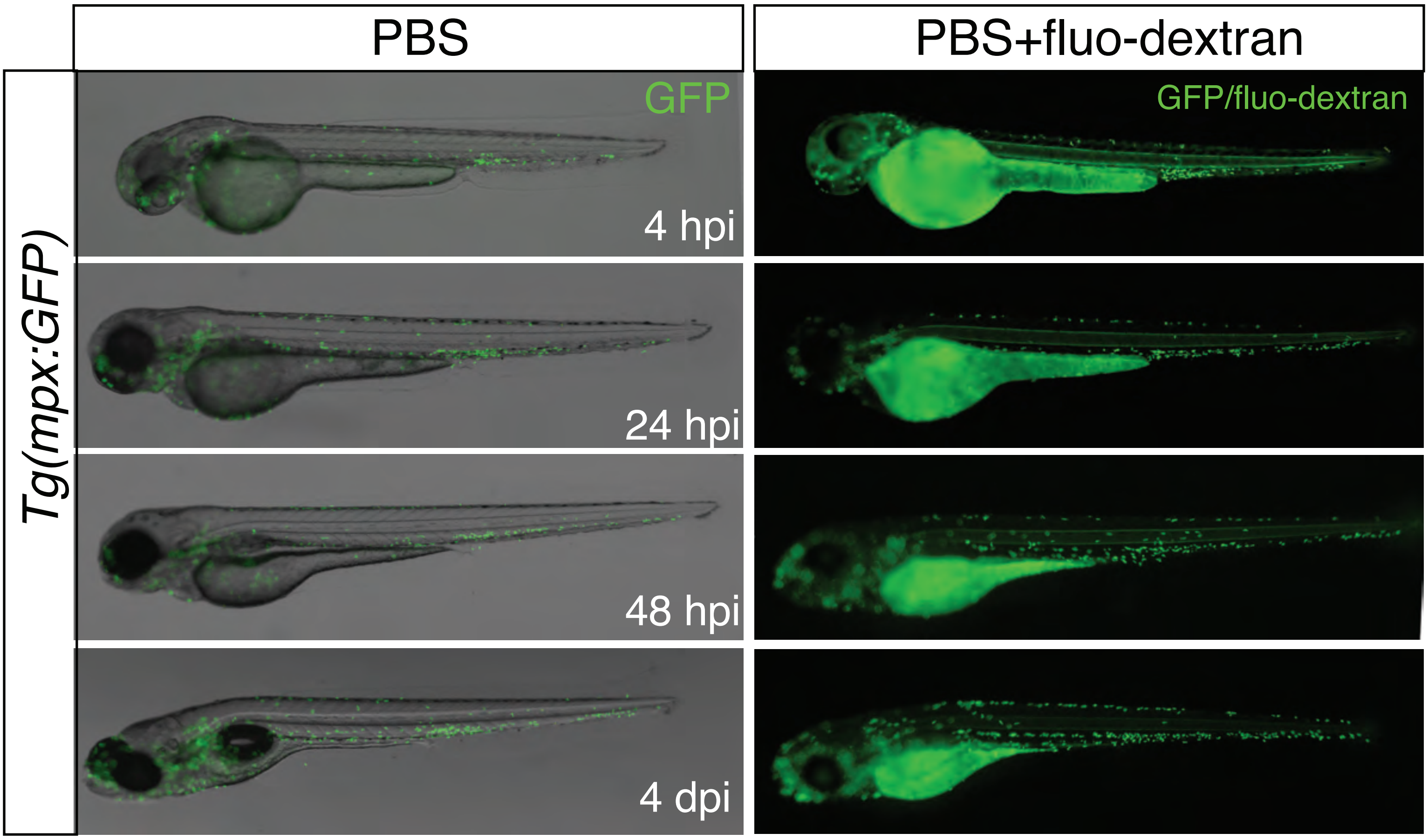

Figure S2: Effect of PBS injection in the notochord on neutrophil recruitment.
To check the effect of the injection in the notochord *per se* on neutrophil recruitment, PBS was injected in the notochord of *Tg(mpx:GFP)* larvae. No neutrophil was recruited to the notochord (visible with bright-field). To confirm and follow the injection in the notochord, PBS combined with Fluorecein-dextran (green) was injected in the notochord of *Tg(mpx:GFP)* larvae. Larvae were imaged at different time points following injection: 4, 24, 48 hpi and 4dpi and no recruited neutrophils (green) was detected.

.

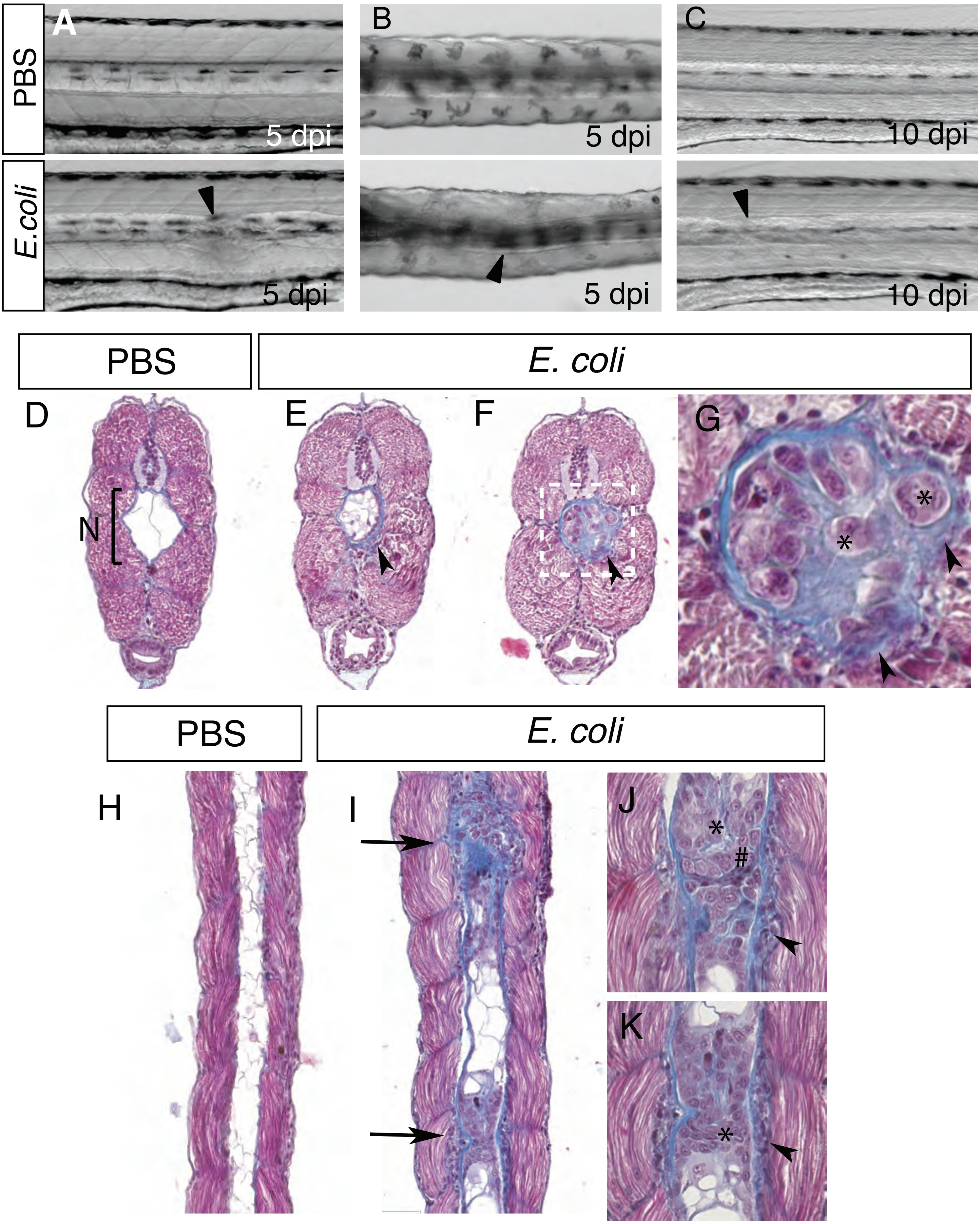

Figure S3: Notochord infection induces notochord defects

Bright-field images of PBS or *E. coli* injected larvae in the notochord at 5 dpi (A-B) and 10 dpi (C). A, C lateral views; B, dorsal view. Arrowheads show defects in the notochord anterior of the injection site (5 to 10 somites away). (D-G) 4 µm cross sections of PBS (D) or *E. coli* (E-G) injected larvae stained with Masson's Trichrome. While PBS injected larvae present a notochord (N, bracket) filled mainly with 2 or 3 big vacuolated cells (white) and delimited with a regular, unaffected collagen membrane (blue), *E. coli* injected larvae present several abnormalities of the notochord. Numerous cells and vacuoles are found inside the notochord. Increase of collagen deposition appears inside and outside the notochord (arrowhead). Many cells are embedded inside the collagen sheath (asterisks). (G) is a high magnification image of the region boxed in (F). (H-K) 4 µm longitudinal sections of PBS (H) or *E. coli* (I-K) infected larvae stained with Masson's Trichrome. As described above, PBS injected larvae present a notochord filled mainly with vacuolated cells (H), while *E. coli* infected larvae present several massive cell aggregates within the notochord (I). In these locations, the notochord is surrounded with cells embedded in the matrix (arrow heads) and cells with different

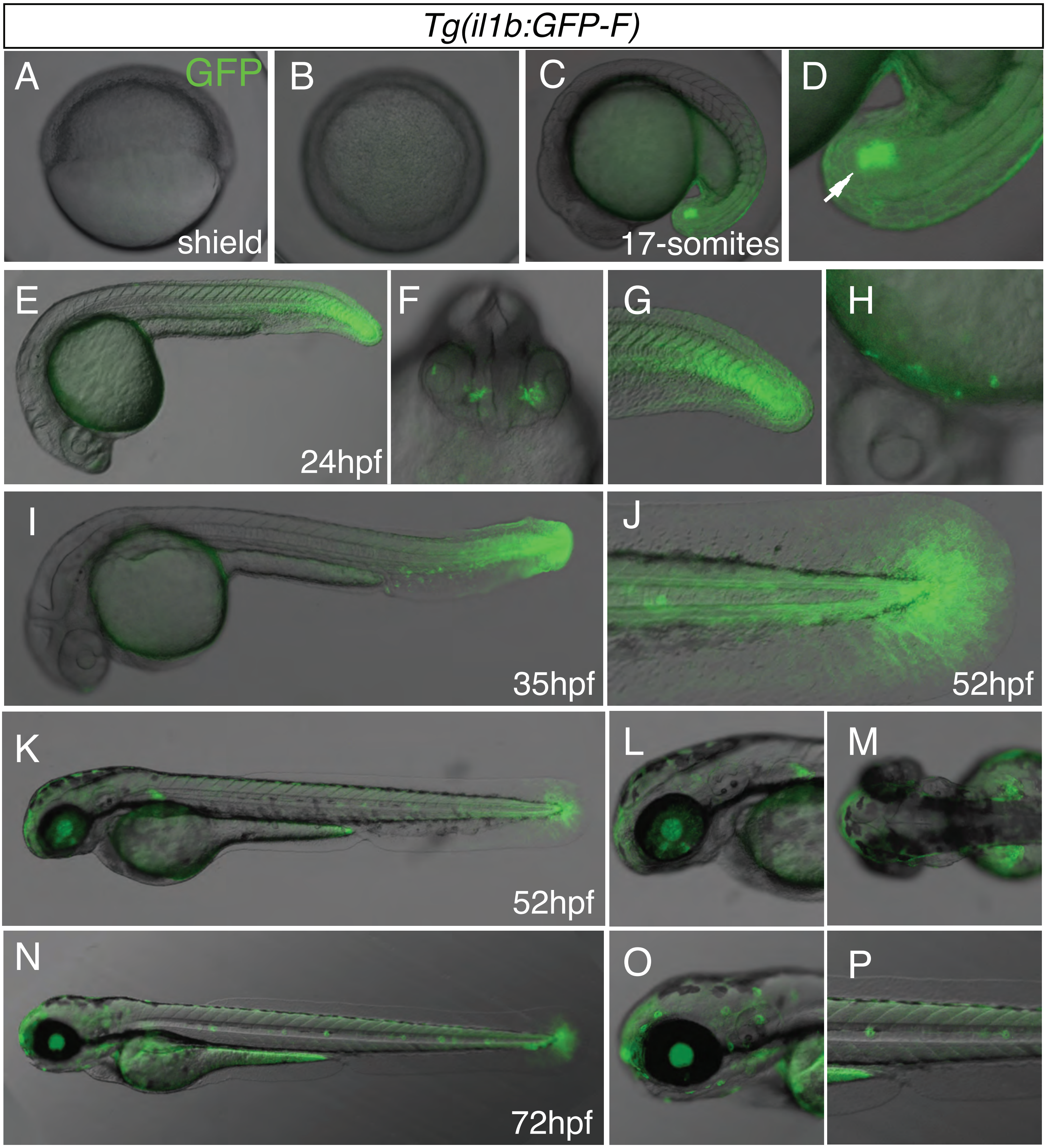

Figure S4: GFP expression in *Tg(il1b:GFP-F)* embryos and larvae during normal development.
(A-B) GFP is not detected during gastrulation: Shield stage, side view with animal pole on top (A) and animal pole view (B). (C-D) GFP is first detected during segmentation in the tail bud, especially in the tip (arrow). (E-I) This expression pattern is seen at 24 and 35hpf, with in addition expression in the olfactory epithelium (F) and in scattered cells on the yolk sac (H). (J-P) From 50 hpf GFP is detected in the tip of the tail and of the caudal fin, especially in the keratinocytes. In addition it is observed in the pectoral fin bud, the retina and the neuromasts. This expression was constant during one month. (C-P) lateral views except (F) (front) and (M) (dorsal).

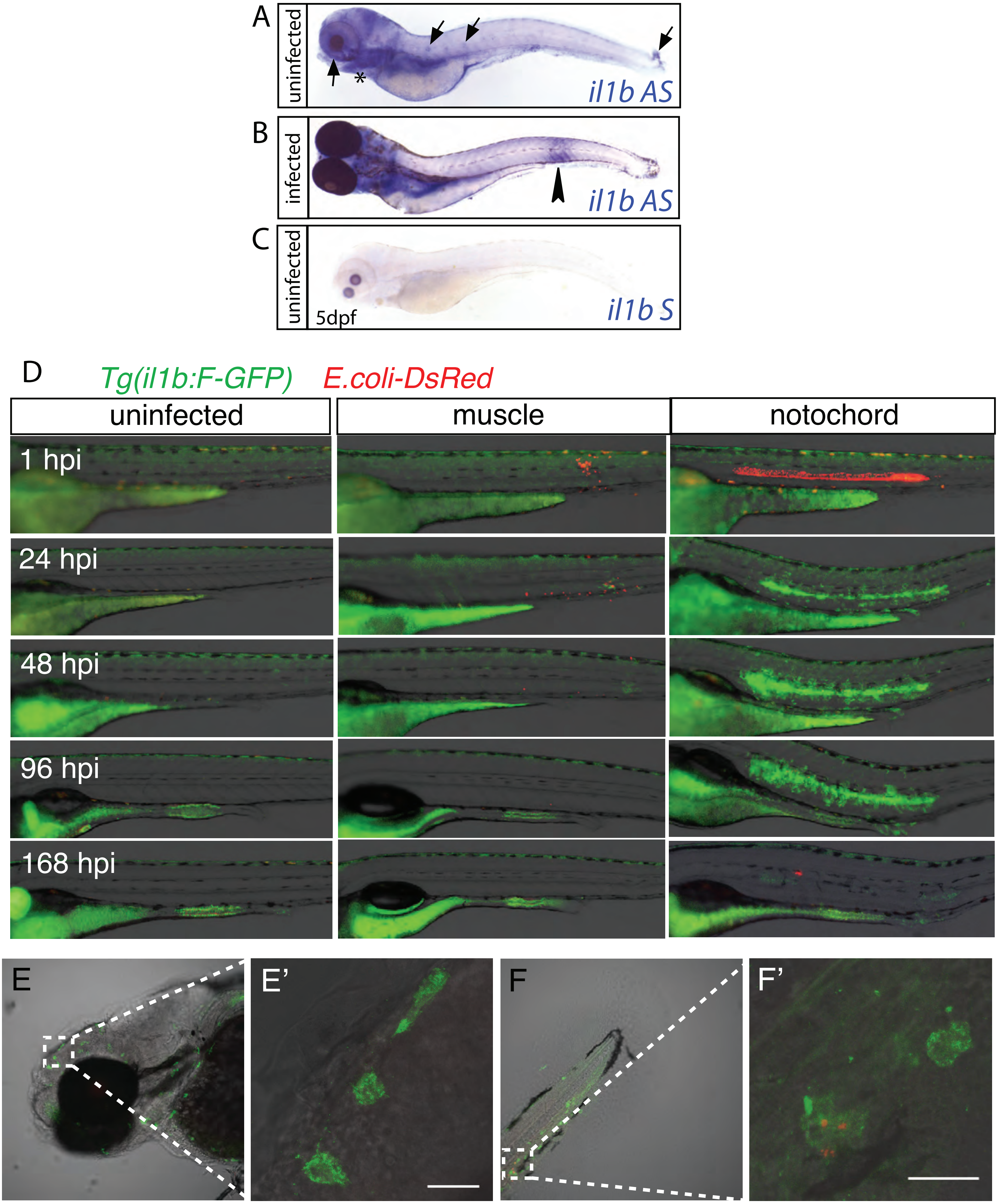

Figure S5: transcriptional activation of *il1b* upon infection with *E. coli*
(A-B) Whole mount in situ hybridization with il1b anti-sense (il1b AS) probe in 5dpf larvae uninfected (A) and infected with *E. coli* in the notochord (3dpi) (B). Probe signal is detected in the tip of the caudal fin, the skin, the neuromast, eyes (arrows) and gills (asterisks). After infection, larvae overexpress *il1b* mRNA in the inflamed region (arrowhead). (C) No signal is detected using il1b sense probe (il1b S) (C). (D) GFP (green) expression in *Tg(il1b:GFP-F)* larvae. Larvae were imaged without infection or at different time points following infection with a DsRed expressing *E. coli* (red) in the muscle or in notochord. Each column shows images from a single larva image repeatedly. (E-F) Confocal analysis of GFP expression in *Tg(il1b:-GFP-F)* in the head (G) and the tail (H) regions 24h following *E. coli* injection. Maximum projections, lateral views, scale bars=20µm. (E') and (F') are high magnification images of regions boxed in E and F.

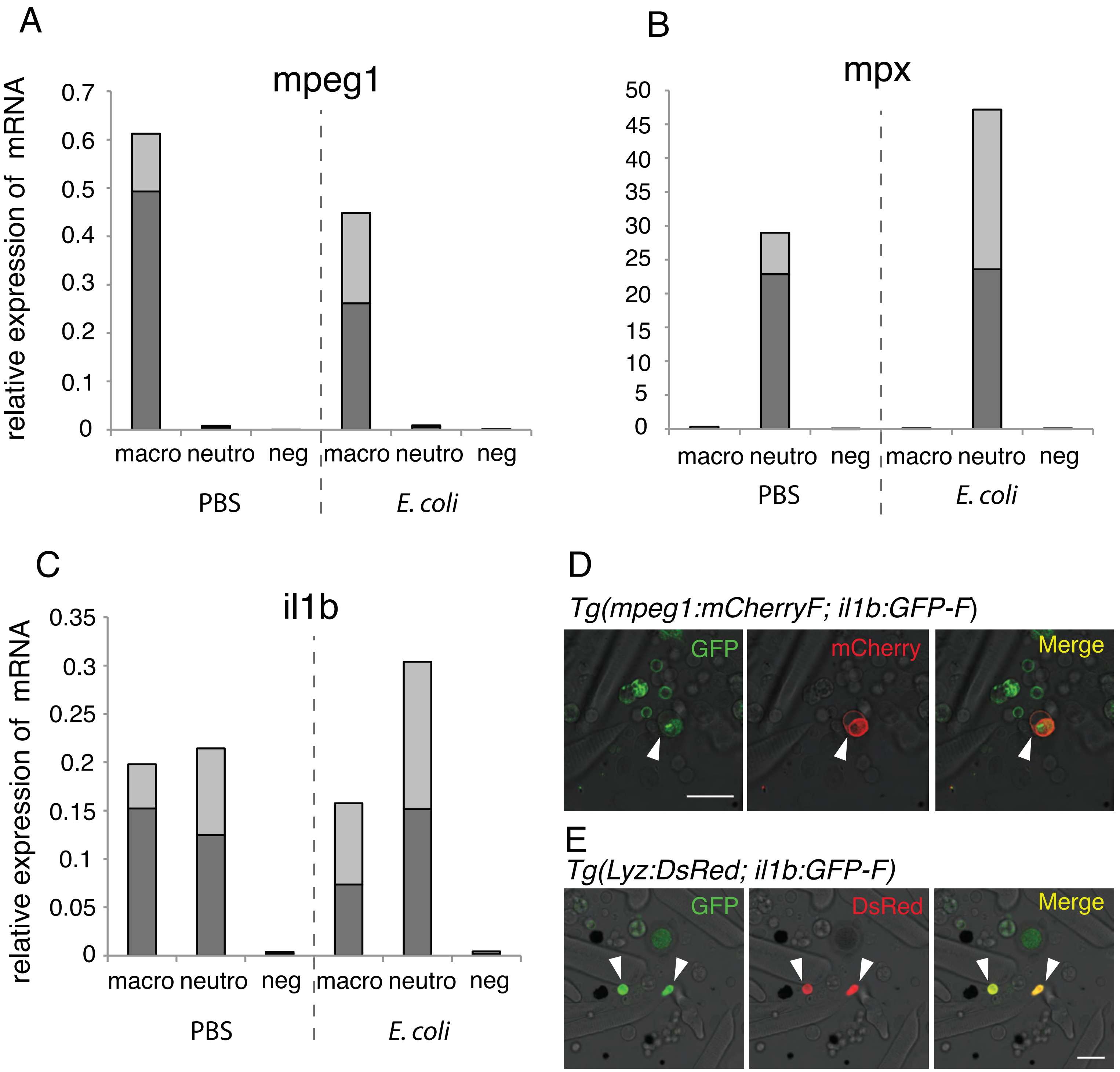

Figure S6 : GFP expression in *Tg(il1b:GFP-F)* line recapitulates endogenous *il1b* mRNA expression in activated leukocytes

Trypsinization of 4dpf larvae was used to generate single cell suspensions for FACS sorting, and was found to activate macrophages and neutrophils, allowing us to compare endogenous *il1b* transcription and GFP expression triggered by a stimulus irrespective of an infection,

(A-C) To test whether *Tg(il1b:GFP-F)* can be used to study the dynamic expression of *il1b* in activated leukocytes, macrophages (macro), neutrophils (neutro), and non leukocyte cells (neg) were FACS sorted from *Tg(mpx:GFP; mpeg1:mCherryF)* 4dpf-larvae first injected with either PBS or *E. coli*. Q-RT-PCR was used to measure the relative expression of *mpeg1* (A) and *mpx* (B) in the different populations, confirming purity of sorted populations. Measurement of *il1b* expression (C) shows that trypinization and cell dissociation of larvae induced high level of *il1b* expression in macrophages and neutrophils as compared to non leukocyte cells in both PBS and *E. coli* injected embryos. Quantifications using *ef1a* as a reference gene; light grey bar represent 95% confidence interval (student T test). Non-overlapping light grey means $p<0.05$.

(D-E) Using similar conditions to dissociate the embryos, leukocytes were cultured from *Tg(il1b:GFP-F; mpeg1:mCherryF)* (D) or *Tg(il1b:GFP-F; Lyz:DsRed)* (E). While no expression of GFP was observed in leukocytes before dissociation (in living larvae, not shown), 90% of the macrophages (red, D) and 100% of the neutrophils (red, E) expressed GFP (green) (arrowheads), showing that *Tg(il1b:GFP-F)* is a good reporter transgene in leukocytes. Confocal images, Scale bar= 20μm

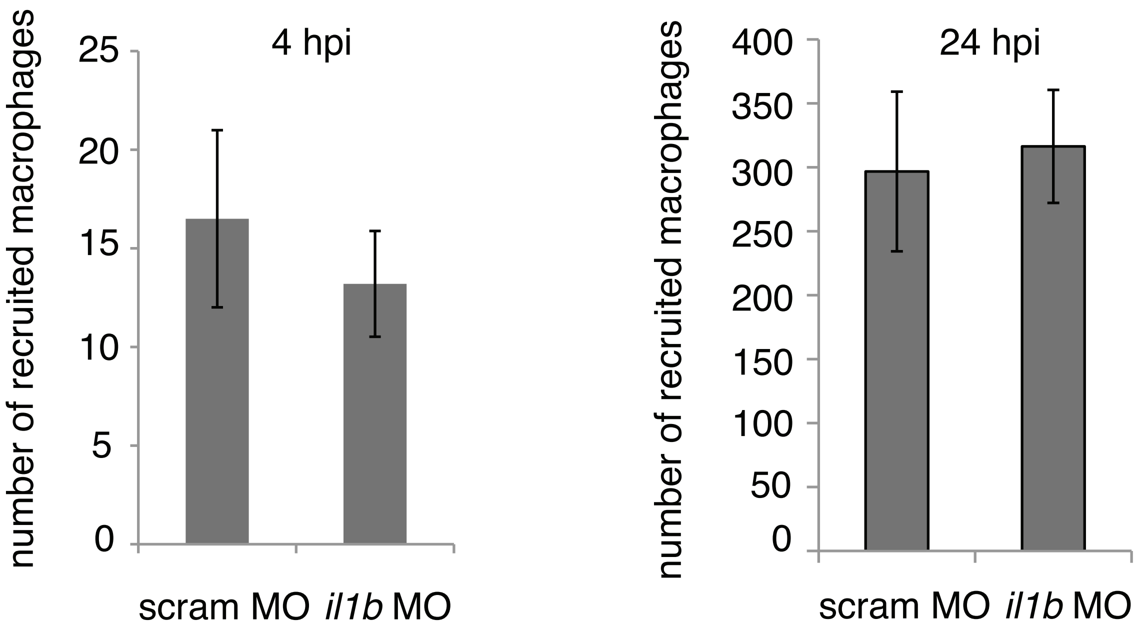

Figure S7: Macrophage recruitment is not affected by *il1b* loss-of-function upon notochord infection. To evaluate the effect of *il1b* loss-of-function on macrophage recruitment, *Tg(mpeg1:mCherryF)* larvae were injected with either *il1b* (il1b MO) or scramble (scram MO) morpholinos. Then the larvae were infected with *E. coli*-GFP in the notochord and recruited macrophages counted. Plots represent the number of recruited macrophages to the notochord at 4 hpi (A) and at 24 hpi (B). The number of recruited macrophages in *il1b* morphans was similar to that of controls. Graphs are mean value ± s.e.m. (A) p=0.80 and (B) p=0.69

	accession number	genes	Fold	s.e.m
increased	NM_212859.2	TNFa1	55.68	0.48
	NM_001024447.1	TNFa2	3.26	1.62
	NM_001020785	Il10	3.44	0.78
unchanged	NM_001082955.1	Il20	1.18	0.07
	NM_001020799	Il26	1.76	0.09
	NM_001111083.1	IFNϕ3	1.57	0.52
	NM_001020792	Il22	1.03	0.05
	XM_002662056.3	CXC11L	0.63	0.30
	NM_131834	CXCR4b	0.75	0.06
decreased	XM_005165411.1	CXC10L	0.40	0.19

Table S1: Induction of cytokines upon notochord infection with E. coli

The relative expression levels of cytokines and chemokines associated with *E. coli* infection in the notochord. Expressions were measured by real-time PCR (using *ef1a* as a reference gene) on cDNA made from RNA isolated from larvae injected with PBS or with *E. coli* in the notochord at 48 hpi. Results are represented as fold change in *E. coli* infection relative to PBS injection +/- standard error of the mean. *n*=3 independent samples of 10 pooled larvae. We detected no significant expression of interferon ϕ1, ϕ2, γ1 and γ2 in the different conditions.

MOVIE LEGENDS

Movie 1: General behaviour of neutrophils following notochord infection

Tg(mpx:GFP) larvae were infected with crimson expressing *E. coli* (magenta) in the notochord at 48hpf. General behaviour of neutrophil (green) was imaged using 4-dimensional confocal microscopy at 2 hpi during 3 hours. Scale bar: 20µm.

Movie 2: General behaviour of neutrophils following notochord infection

Tg(mpx:GFP) larvae were infected with DsRed expressing *E. coli* (red) in the notochord at 48hpf. Scale bar: 50µm. Neutrophil behaviour (green) was imaged at 1 hpi during 2 hours and 50 min.

Movie 3: Typical behaviour of one neutrophil following notochord infection

High magnification of a region of movie S2 shows the representative behaviour of one neutrophil that fail to engulf bacteria. Scale bar: 20µm.

Movie 4: General behaviour of macrophages following notochord infection

Tg(mpeg1:mCherryF) larvae were infected with GFP expressing *E. coli* (green) at 48hpf in the notochord. The behaviour of macrophages (red) was imaged using 4-dimensional confocal microscopy at 1 hpi during 3 hours. Scale bar: 50µm.

Movie 5: Typical behaviour of few macrophages following notochord infection

The representative behaviour of few macrophages was image in a similar experiment that describe in movie S5 from 1hpi during 3 hours 20 min. Scale bar: 20µm.

Movie 6: General behaviour of macrophages following systemic infection

Tg(mpeg1:mCherryF) larvae were infected with GFP expressing *E. coli* (green) at 48hpf in the posterior caudal vein. The behaviour of macrophages (red) was imaged using 4-dimensional confocal microscopy at 1 hpi during 1 hour. Scale bar: 50μm.

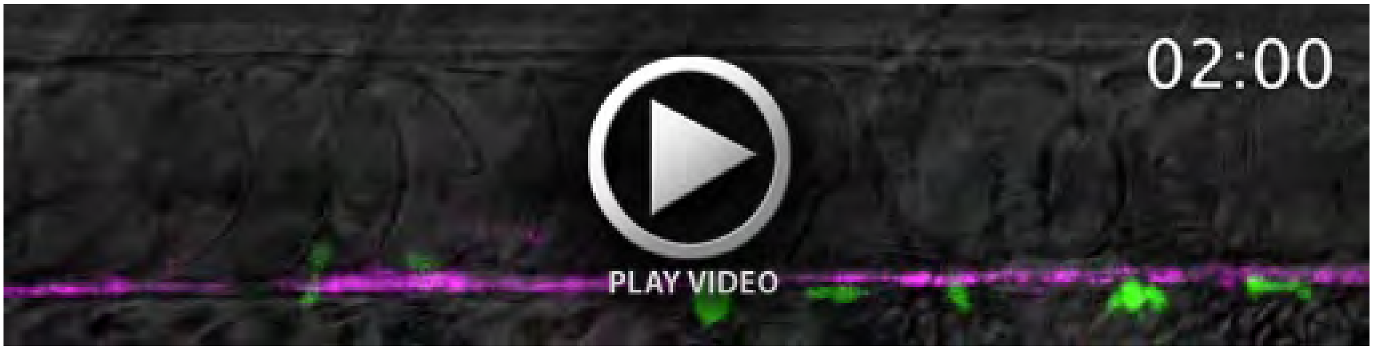

Movie 1.

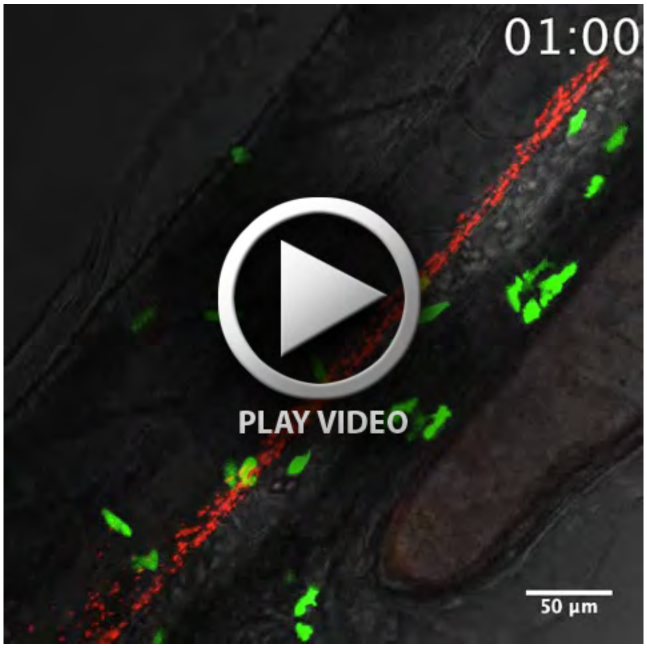

Movie 2.

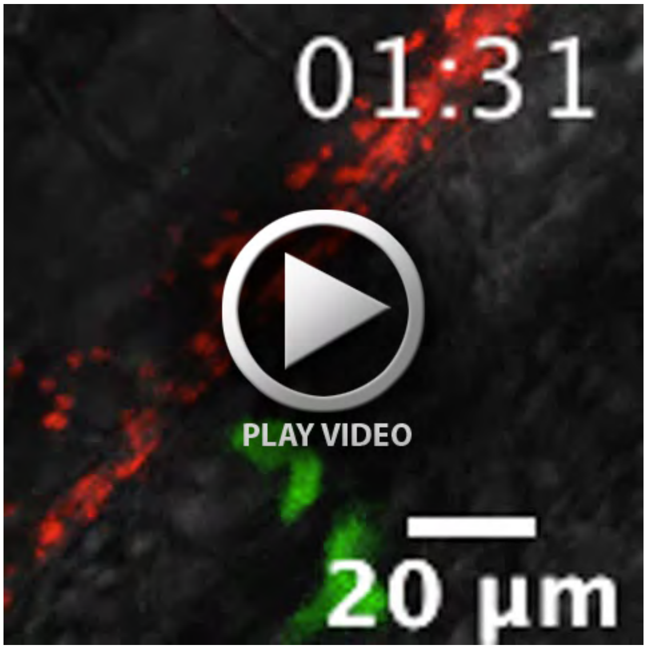

Movie 3.

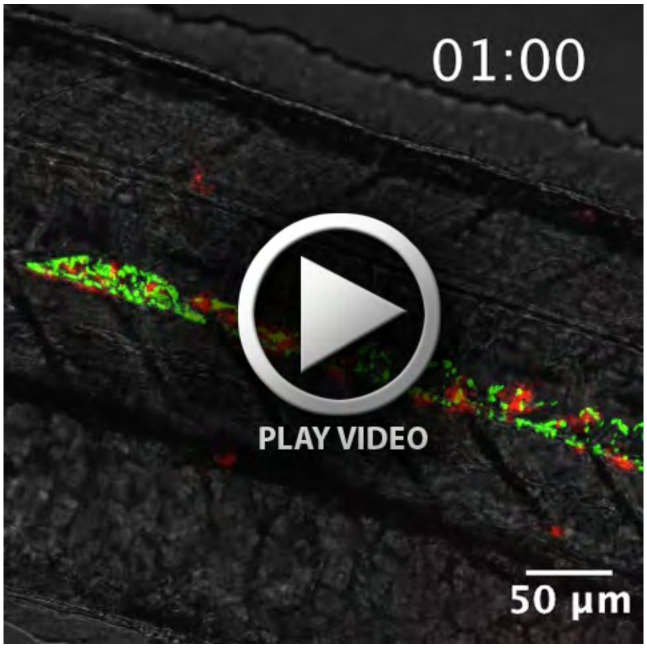

Movie 4.

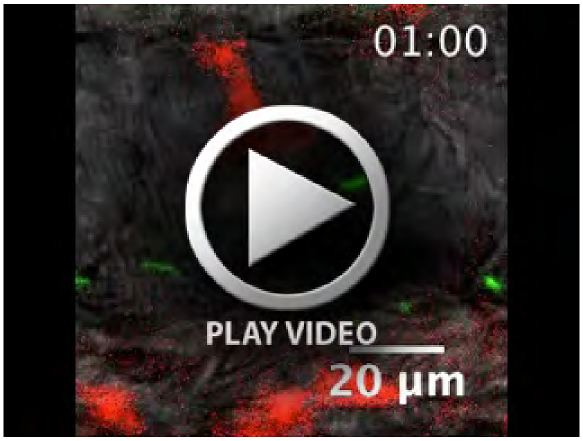

Movie 5.

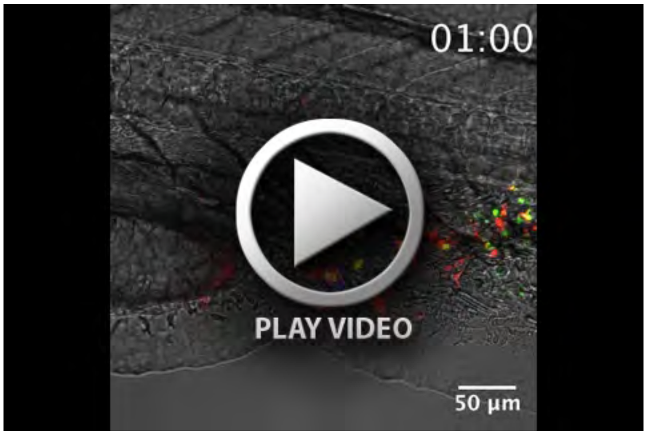

Movie 6.
